# Targeted enrichment and high-resolution digital profiling of mitochondrial DNA deletions in human brain

**DOI:** 10.1111/acel.12146

**Published:** 2013-09-11

**Authors:** Sean D Taylor, Nolan G Ericson, Joshua N Burton, Tomas A Prolla, John R Silber, Jay Shendure, Jason H Bielas

**Affiliations:** 1 Translational Research Program, Public Health Sciences Division, Fred Hutchinson Cancer Research Center1100 Fairview Ave, Seattle, WA, 98109, USA; 2 Department of Genome Sciences, University of Washington3720 15th Ave NE, Seattle, WA, 98195, USA; 3 Department of Medical Genetics, University of Wisconsin-Madison425-G Henry Mall, Madison, WI, 53706, USA; 4 Neurological Surgery, University of Washington Medical Center1959 NE Pacific St, Seattle, WA, 98195, USA; 5 Human Biology Division, Fred Hutchinson Cancer Research Center1100 Fairview Ave, Seattle, WA, 98109, USA; 6 Department of Pathology, University of Washington Medical Center1959 NE Pacific St, Seattle, WA, 98195, USA

**Keywords:** aging, genome instability, mitochondrial disease, mitochondrial DNA, next-generation sequencing, rare deletion detection

## Abstract

Due largely to the inability to accurately quantify and characterize *de novo* deletion events, the mechanisms underpinning the pathogenic expansion of mtDNA deletions in aging and neuromuscular disorders remain poorly understood. Here, we outline and validate a new tool termed ‘Digital Deletion Detection’ (3D) that allows for high-resolution analysis of rare deletions occurring at frequencies as low as 1 × 10^−8^. 3D is a three-step process that includes targeted enrichment for deletion-bearing molecules, single-molecule partitioning of genomes into thousands of droplets for direct quantification via droplet digital PCR, and breakpoint characterization using massively parallel sequencing. Using 3D, we interrogated over 8 billion mitochondrial genomes to analyze the age-related dynamics of mtDNA deletions in human brain tissue. We demonstrate that the total deletion load increases with age, while the total number and diversity of unique deletions remain constant. Our data provide support for the hypothesis that expansion of pre-existing mutations is the primary factor contributing to age-related accumulation of mtDNA deletions.

## Introduction

The human mitochondrial genome is a small (16.5 kb) circular DNA molecule that is present in multiple copies per cell (between 1000 and 10 000 copies depending on the cell type) (Berdanier & Everts, 2001). This small genome is densely packed with 13 structural genes that encode the major catalytic components of the core complexes involved in oxidative phosphorylation (OXPHOS), as well as 22 tRNAs and 2 rRNAs that are essential for mitochondrial protein synthesis (Scheffler, 2008). Because of the density of the gene structure, deletions in mitochondrial DNA (mtDNA) tend to affect multiple genes, including several essential tRNAs.

Accumulated mitochondrial deletions are known to cause a number of neuromuscular disorders, including Kearns–Sayre syndrome, progressive external ophthalmoplegia, and Pearson syndrome (Chinnery, 1993; Berdanier & Everts, 2001; Greaves *et al*., 2012). These diseases are typically (but not exclusively) associated with a 4977-bp ‘common’ deletion between np 8482 and np 13 460. Additionally, an increasing number of associations are being discovered between mtDNA and cancer. Cancer-associated deletions tend to be smaller (< 1 kb) than those associated with neuromuscular disorders (Lee *et al*., 2010). Whereas accumulation of large deletions leads to mitochondrial dysfunction and apoptosis, it is thought that small deletions may confer milder phenotypes that can promote tumor cell proliferation, drug resistance, and malignancy. Finally, accumulation of mtDNA deletions in postmitotic tissue (e.g., brain, heart, and skeletal muscle) is thought to be an important driving force in both physiological and accelerated aging (Cortopassi & Arnheim, 1990; Meissner *et al*., 2008; Vermulst *et al*., 2008b; Khrapko & Vijg, 2009).

In neuromuscular disorders, cancer and aging, the pathological mtDNA deletions appear to be somatically acquired (Meissner, 2007; Meissner *et al*., 2008). Furthermore, individual mitochondrial mutations must expand above a threshold intracellular frequency, typically 60–90% of a cell’s mtDNA, before it reaches phenotypic expression (Vermulst *et al*., 2012). Thus, the etiology of mitochondrial deletion diseases necessarily involves two distinct processes: the somatic generation of the deletion(s) and their subsequent expansion to phenotypic levels. However, neither of these processes is well understood (Krishnan *et al*., 2008; de Grey, 2009; Song *et al*., 2011). One of the key difficulties is a lack of sensitive assays to detect *de novo* deletions, which in normal tissue may be lower than 1 deletion per million genomes, and track the kinetics of their initial selection. Current assays lack the sensitivity to capture these rare events without first amplifying the target sites, typically via PCR (Cortopassi & Arnheim, 1990; He *et al*., 2002; Chabi *et al*., 2003; Kraytsberg *et al*., 2008). This practice is subject to introduction of numerous artifacts, is biased toward amplification of large products, and often only allows detection of a subset of deletions that have already undergone some level of expansion. The increasingly large body of work devoted to elucidating the mechanisms by which somatically acquired deletions undergo intra- and/or intercellular expansion serves to underscore the need for more sensitive tools to study this important phenomenon (Cortopassi & Arnheim, 1990; Coller *et al*., 2001; Foury *et al*., 2004; Durham *et al*., 2006; Krishnan *et al*., 2008; Fukui & Moraes, 2009; Kato *et al*., 2011; Payne *et al*., 2011; Song *et al*., 2011; Freyer *et al*., 2012; Vermulst *et al*., 2012).

To more sensitively characterize the formation and expansion of mitochondrial deletions, we have developed a new procedure for quantitative analysis of rare deletion events. This assay, termed ‘Digital Deletion Detection’ (3D), allows us to directly quantify and characterize site-specific rare mitochondrial deletions that occur at frequencies as low as 1 deletion per 100 million genomes. We demonstrate that 3D is accurate over a broad dynamic range and is capable of detecting both specific and random deletion events within a targeted region of the mitochondrial genome. We have successfully used 3D to study accumulation of clonal and random mitochondrial deletions in human brain tissue with respect to age, allowing a high-resolution analysis of deletion dynamics in aging tissue.

## Results

### Assay design

**Figure 1:**
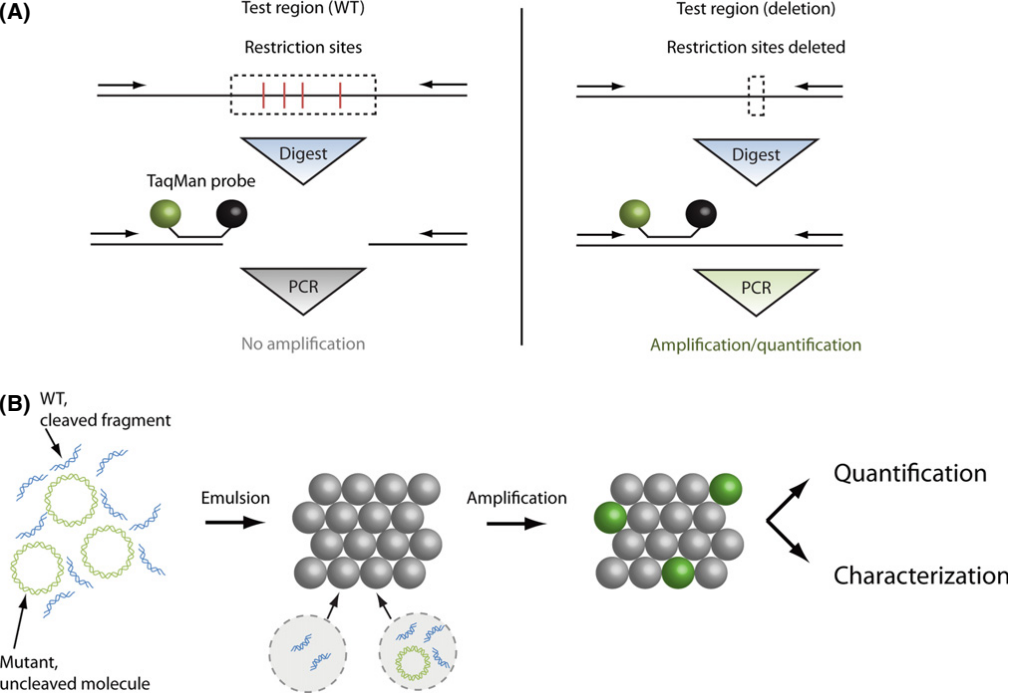
Overview of Digital Deletion Detection (3D). (A) Enrichment of deletion-bearing molecules. WT molecules harbor endonuclease recognition sites within the target region. Upon digestion, the target is cleaved, making the WT molecule unsuitable as a template for PCR amplification. In contrast, mutant molecules that harbor deletions and remove the restriction recognition sites are resistant to digestion. These molecules serve as templates for PCR amplification. The presence of the TaqMan® hydrolysis probe allows for the detection and enumeration of each molecule in the sample bearing the appropriate deletion. (B) Mutant target molecules (depicted as an idealized, unbroken circular mitochondrial chromosome) are individually sequestered into 1 nL water-in-oil droplets along with TaqMan® PCR chemistry and target-specific TaqMan® probes. Droplets are thermally cycled. Because the average number of molecules per droplet is less than one, positive droplets (green droplets) represent individual reaction vessels for single-molecule quantitative PCR amplification. Droplets are individually scanned and scored as positive or negative, thus providing a digital quantification of all deletion-bearing molecules within the sample. Alternatively, droplets can be disrupted and the amplification products subjected to physical characterization, for example cloning, sequencing, or other applications.

Digital Deletion Detection (3D) is an extremely sensitive tool for the absolute quantification and characterization of rare deletion molecules. The basic strategy behind 3D is a three-step process: enrich, amplify, and analyze. The first step, based on methods developed previously by Bielas and colleagues, enriches for deletion-bearing molecules and improves mutant specificity (Bielas & Loeb, 2005; Vermulst *et al*., 2008a). This step consists of targeted endonucleolytic digestion of templates to selectively digest wild-type (WT) molecules, thus allowing the preferential PCR amplification of molecules bearing an appropriate deletion (Fig. 1A). After digestion, the DNA molecules are sequestered into homogenous 1 nL water-in-oil emulsion droplets and subjected to normal PCR amplification (Fig. 1B). The concentration of molecules within the droplets is adjusted such that most droplets contain no mutant genomes, while a small fraction contains only one. Thus, a single well in the reaction actually consists of many thousand single-molecule reaction chambers. This process allows each captured deletion to be amplified without bias and without introducing many of the PCR artifacts that are common to bulk amplification reactions (i.e., template switching and preferential amplification of short templates).

Following amplification, the deletions can be analyzed via two process pathways. In the quantification pathway, high-resolution quantification of deletions is accomplished through the use of droplet digital PCR (ddPCR) (Pinheiro *et al*., 2012). With the inclusion of TaqMan reporter chemistry, droplets bearing amplified templates are readily distinguished by their fluorescence amplitude using a cytometry system. Because the droplet volumes are highly uniform, Poisson statistics can be applied to calculate the average number of deletion-bearing molecules per droplet and the absolute concentration of mutant molecules determined with high precision and accuracy (Pinheiro *et al*., 2012). Alternatively, in the characterization pathway, droplets are disrupted and amplicons recovered. The deletions can then be directly sequenced using high-throughput or ‘next-generation’ sequencing or cloned for use in Sanger sequencing or other downstream applications.

### Sensitivity and recovery

**Figure 2:**
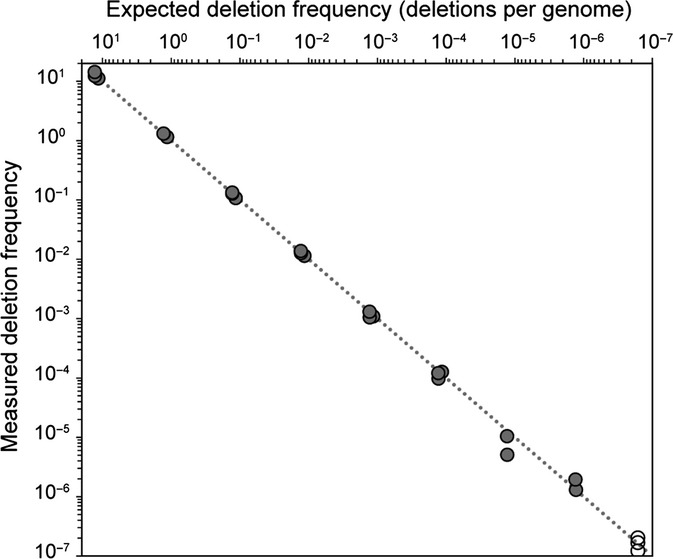
Sensitivity and recovery. 3D was performed on *TaqI*-digested HCT 116 mtDNA using primers and probes for the human ND1/ND2 site to give the endogenous deletion frequency (empty circles). Reconstruction experiments were performed by spiking in 3 molecules μL^−1^ of a control plasmid bearing a portion of the human mitochondrial genome with a known deletion (3534Δ997) into a serial dilution series of *TaqI*-digested HCT 116 mtDNA (filled circles). The predicted deletion frequency is plotted against the measured deletion frequency. Each data point represents an individual experiment. The reconstruction data were fit to y = x (dotted line) with a correlation coefficient *R*^2^ = 0.9942.

Using the quantification process pathway of 3D (Fig. 1), we measured the absolute deletion frequency within a region spanning the ND1/ND2 genes in mitochondrial DNA isolated from human epithelial cells in tissue culture. We measured the deletion frequency to be 1.6 ± 0.4 deletions per ten million genomes (or 1.6 × 10^−7^ per genome) (Fig. 2). We next asked whether 3D was able to fully recover all of the deletions within a sample over a broad range of deletion frequencies. To address this, we performed a series of reconstruction experiments. First, a plasmid harboring a fragment of mtDNA containing a known deletion in the ND1/ND2 region was mixed at a constant concentration (3 copies μL^−1^) against increasingly higher levels of genomic mtDNA (up to 2.5 × 10^6^ copies μL^−1^). We then performed 3D analysis to determine whether the small concentration of the control molecules could be accurately quantified in the presence of increasing concentrations of background DNA (Fig. 2). This reconstruction demonstrated accurate quantification of target molecules across a range of frequencies spanning eight orders of magnitude, with sensitive recovery at frequencies as low as 1 × 10^−7^ per genome.

Because we reached the endogenous deletion frequency of the background DNA, we were unable to test lower frequencies in the reconstruction experiment. To determine whether we could detect even rarer events, we applied 3D to mtDNA isolated from muscle samples of mice, choosing a site encompassing the light chain origin of replication (Supplementary Note 4). Because deletion of this site would severely impede the ability of the genome to replicate, we expected the deletion frequency at this site to be extremely low. 3D analysis revealed a deletion frequency of 1.3 ± 0.4 × 10^−8^ per genome (Fig. S3).

### Capturing and analyzing sample complexity

**Figure 3:**
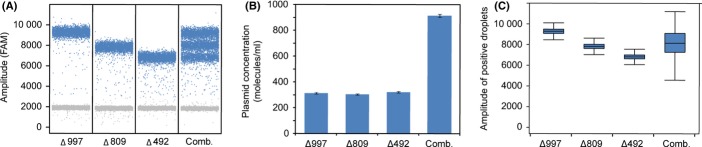
Effects of sample heterogeneity on 3D analysis. (A) Three plasmid controls (3534Δ997, 3719Δ809, and 3871Δ492) were diluted to an expected concentration of 300 molecules μL^−1^ template and subjected to 3D analysis, either individually or combined. Blue dots represent droplets whose amplitudes are above the threshold (‘positives’), while gray droplets are those whose amplitudes are below the threshold value (‘negatives’). (B) Measured deletion concentration for individual and combined templates. Error bars indicate the Poisson 95% confidence intervals for each concentration determination. (C) Box and whisker plot showing the distribution of positive droplets for each template. When used as a template in PCR, each plasmid yields different size fragments (185 bp, 372 bp, and 686 bp, respectively). There is an inverse relationship between average fluorescence amplitude and template length, as well as a relationship between the sample complexity and the breadth of the distribution of positive droplets.

Next we characterized the ability of 3D to perform accurate quantification of the deletion frequency when applied to a heterogeneous population of deletions. To this end, we obtained three control plasmids, each containing an mtDNA fragment harboring a unique deletion from the minor arc of the human mitochondrial genome (3534Δ997, 3719Δ809, and 3871Δ492). We subjected equal amounts (300 molecules μL^−1^) of each control plasmid to 3D analysis, either separately or combined into a single reaction, to determine whether 3D could accurately report the known concentration of a mixture of target molecules (Fig. 3A). 3D quantification of the individual plasmids yielded concentrations of 313 ± 6, 304 ± 6, and 322 ± 6 molecules μL^−1^, respectively (Fig. 3B). Quantification of the combined reaction yielded a concentration of 915 ± 12 molecules μL^−1^. These values match the expected concentrations within the limits of uncertainty due to the stochastic effect associated with sampling of a dilute solution (Pinheiro *et al*., 2012).

Analysis of fluorescence amplitudes of the three control plasmids following ddPCR revealed that under the current conditions, a given template will yield an average droplet fluorescence intensity inversely proportional to the template size (Fig. 3C). When the three control templates were combined, this effect led to a striking multimodal distribution in the fluorescence amplitudes (Fig. 3A). More generally, we found that the sample heterogeneity is reflected in the distribution of fluorescence amplitudes (Fig. 3C). Thus, the average amplitude and distribution of the droplet fluorescence can be used to predict deletion sizes and complexity (e.g., presence of a single, clonal deletion vs. a heterogeneous population of multiple deletions).

### Deletion dynamics in aging postmitotic tissue

**Figure 4:**
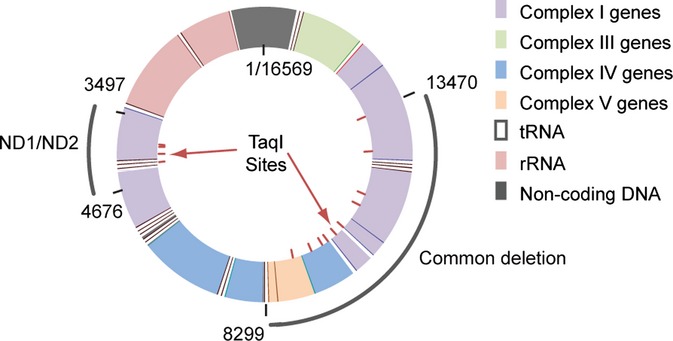
Deletion sites for 3D analysis of brain mitochondrial DNA. Probe and primer sets were designed to detect deletions in two regions of the human mitochondrial genome. The first region is defined by a primer set that flanks np 8299–13470 and is designed to detect variants of the common deletion. The second primer set flanks np 3497–4676, spanning the junction between the ND1 and ND2 genes in the minor arc. The common deletion primer set flanks ten *TaqI* sites, while the ND1/ND2 primer set flanks four *TaqI* sites.

**Figure 5:**
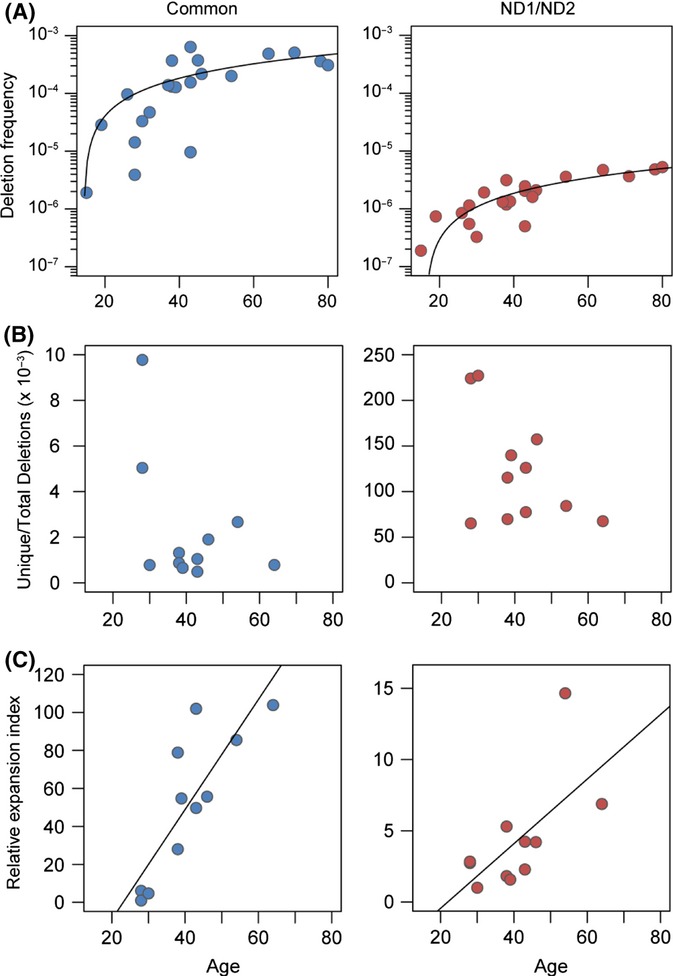
3D analysis of deletion frequency in aged human brain tissue. (A) Total deletion frequency at each site is plotted against age. Deletion frequency shows a positive correlation with age at both the common deletion site (*R*^2^ = 0.453, *P* = 0.0008) and the ND1/ND2 site (*R*^2^ = 0.812, *P* = 3 × 10^−8^). The linear regression (transformed) is shown on a log-scale plot. (B) Deletion profiling was performed on a subset of patients to examine the diversity of deletions present in the deletion pool. The number of unique deletions is shown normalized against the total number of deletions for each patient in the subset, respectively. At the common deletion site, patients showed a range of 0.5–10 unique per 1000 deletions. At the ND1/ND2 site, the deletion diversity was much higher, ranging from 65 to 227 per thousand. Linear regression analysis showed no significant correlation between the unique-to-total deletion ratio and age at either site (*P* = 0.15 and *P* = 0.12 for the common and ND1/ND2 deletion sites, respectively). (C) The relative expansion index for each patient in the subset was found by taking the ratio of total deletion frequency over the number of unique deletions normalized against the youngest time point. This value gives an estimate of the average frequency of individual deletions for each patient relative to the youngest time point (i.e., the average individual deletion frequency). Linear regression showed a positive correlation for both the common deletion site (*R*^2^ = 0.697, *P* = 0.001) and the ND1/ND2 site (*R*^2^ = 0.421, *P* = 0.03).

While it is known that mtDNA deletions accumulate to relatively high levels in aged, postmitotic tissue in humans (Cortopassi & Arnheim, 1990), very little is known about the underlying dynamics. Specifically, as a tissue ages and accumulates deletions, it is unknown whether this increased deletion load arises through clonal expansion of an existing pool of mtDNA deletions (early acquisition), continual accumulation of new mutations (late acquisition), or an equilibrium of both processes (Khrapko, 2011). With 3D, we can now begin to directly assess these longitudinal changes. We used 3D to characterize deletions with respect to age at two regions of the mitochondrial genome from a collection of human brain tissue (Fig. 4). Using the quantification process pathway of 3D, we found that the total deletion frequency increases with age at both sites (Figs 5A and S4). The common deletion was found to gain in frequency from 1.91 ± 0.15 × 10^−6^ per genome at age 15 years to levels as high as 6.36 ± 0.20 × 10^−4^ per genome by age 80 years, an increase of over 300-fold (Table 1). These levels and accumulation rates are in agreement with previously published results (Meissner *et al*., 2008). At the ND1/ND2 site, the absolute levels of accumulation also increased, but were generally lower than at the common deletion site. Deletion frequencies ranged from 1.9 ± 0.5 × 10^−7^ per genome to 5.25 ± 0.22 × 10^−6^ per genome, about a 25-fold increase over the same age span (Table 1). Interestingly, the increase in deletion frequency at the ND1/ND2 site showed a stronger correlation with age than the common deletion site (*R*^2^ = 0.812 vs. 0.453, respectively) (Fig. 5A).

**Table 1:** Frequencies of mitochondrial deletion events in human brain. The error of duplicate measurements is indicated as the standard error of the mean (SEM)

		Measured deletion frequency (× 10^−7^)				Unique deletions (per 1000 total)	
ID	Age	ND1/ND2	SEM	Common	SEM	ND1/ND2	Common
P01†	28	11.3	1.1	141.5	4.2	65.2	5.0
P02†	28	5.2	0.1	38.7	4.2	224.0	9.8
P03†	43	20.0	1.7	6355.2	61.0	126.1	0.5
P04†	30	3.3	2.9	335.8	314.6	227.0	0.8
P05†	38	11.7	1.2	1883.5	556.8	115.3	1.3
P06†	38	31.1	0.1	3687.8	28.6	69.8	0.9
P07†	39	13.0	3.7	1276.0	19.7	139.7	0.7
P08†	43	23.9	0.1	1550.5	5.1	77.5	1.0
P09†	46	20.3	1.2	2174.5	16.2	157.3	1.9
P10†	54	35.3	0.4	1996.7	115.5	84.3	2.7
P11†	64	46.0	1.5	4856.7	162.3	67.6	0.8
P12	43	5.0	0.1	96.0	3.1		
P13	37	13.1	0.2	1389.2	14.8		
P14	19	7.3	1.1	285.8	13.3		
P15	32	19.1	1.7	470.0	5.7		
P16	15	1.9	0.5	19.1	1.5		
P17	45	16.0	0.2	3739.3	154.1		
P18	26	8.4	2.0	961.2	42.3		
P19	80	52.5	2.2	3084.8	20.0		
P20	78	48.0	6.3	3596.7	5.6		
P21	71	36.5	4.1	5069.5	130.5		

† Used in NGS analysis.

To determine whether the increases in deletion frequency at these sites were due to expansion of existing deletions or acquisition and accumulation of new deletions, we sought to measure the ratio of unique to total deletions as a function of age. To accomplish this, emulsion droplets for a subset of patients (*n* = 11) were disrupted and the enriched mutant fragments recovered. We then performed high-throughput massively parallel sequencing analysis on each collection of amplified targets. In this way, we were able to directly profile the entire population of amplified deletion fragments at high resolution. From these data, we were able to determine the total number of unique deletion events present per sampled patient, which was then normalized against the total number of deletions in the sample as measured by ddPCR (Table 1, Fig. 5B). Linear regression analysis showed no significant correlation between the ratio of unique to total deletions and age at either site (*P* = 0.120 and *P* = 0.150 for the ND1/ND2 and common deletion sites, respectively). To ensure that our data are not influenced by sampling or processing artifacts, we analyzed a number of parameters, including the total number of genomes isolated and screened, the number of droplets used in ddPCR analysis, and site saturation effects (Data S1, Supplementary Note 5). Analysis of these parameters indicates that our data are free from any such confounding effects that might artificially skew our results (Figs S4, S5, and S6).

**Figure 6:**
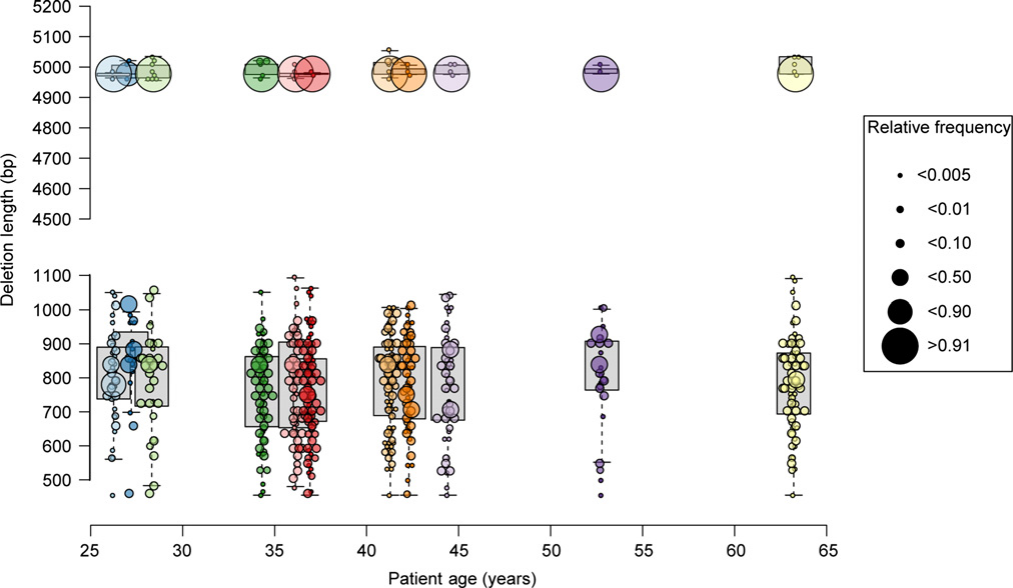
High-resolution analysis of deletion dynamics. Density dot plots showing the length distribution (y-axis) and relative frequency (point size) of all unique deletions per patient in the sequenced subset (x-axis, plotted as age). Box plots and whiskers plots (gray) in the background show the 95% confidence interval of the unweighted length distribution. The common deletion site is shown on the top plot, and the ND1/ND2 deletion site is shown on the bottom plot.

We next analyzed how the diversity within the pool of deletions might change with respect to age. Analysis of the amplitude distribution of positive droplets from ddPCR predicts that there is low heterogeneity at the common deletion site and high degree of heterogeneity at the ND1/ND2 site (Fig. S7). However, at both sites, the diversity does not appear to change with age. These findings were confirmed through breakpoint analysis of the sequenced deletions. Each unique deletion was individually analyzed and characterized by deletion length and relative frequency in the deletion pool (Fig. 6, Data S2). At the common deletion site, we observed a single dominant deletion in every case, which contributed to over 90% of the deletion load (Figs 6 and S8). Although several minor variants are present in each patient, most generally contributed < 0.5% of the total deletion burden. At the ND1/ND2 site, however, there is a broad but fairly uniform distribution of deletion sizes within the ND1/ND2 deletion space across individuals of all ages (Fig. 6, bottom panel). The bulk of the deletion load was typically comprised of deletions which individually contributed between 1 and 10% to the total deletion burden (Figs 6 and S8). The data indicate no major shift in the size distribution of deletions as well as the relative pools of high- and low-frequency deletions with age (Figs 6 and S7).

Finally, we examined the average frequency of individual deletions with respect to age. This was found by taking the ratio of the deletion frequency and the total number of unique deletions, a value we term the expansion index, which is then normalized against the youngest time point for clarity. A decrease in the normalized expansion index with respect to time denotes that deletions are being selected against, while an increase suggests positive selective pressure. We found that at both sites, the expansion ratio increases significantly with age (Fig. 5C). Concomitant with a static spectrum of deletion diversity with age, we conclude that expansion of a pre-existing set of deletions may be one of the primary drivers of age-related increases in deletion frequency.

## Discussion

To adequately detect *de novo* mtDNA deletions and trace the frequency dynamics, an assay is needed that can enrich for and directly quantify extremely rare deletion events. Current approaches to analyzing mtDNA deletions include Southern blotting (DiMauro & Hirano, 1993), direct sequencing (Spelbrink *et al*., 2000; Ameur *et al*., 2011; Kato *et al*., 2011; Sequeira *et al*., 2012), and PCR amplification (Kraytsberg *et al*., 2008). Sequencing of deletions via cloning is laborious, time-consuming, prone to cloning artifacts and allows only the most abundant deletion types to be analyzed (Supplementary Notes 3 and 4). Massively parallel or ‘next-generation’ sequencing is rapidly becoming a preferred means for high-throughput screening of individual DNA molecules. As an example, Illumina, Inc. (San Diego, CA, USA) offers systems that generate from 17 million (MiSeq®) up to 3 billion simultaneous sequencing reads per run (HiSeq®) (Liu *et al*., 2012). However, given a relatively short read length of < 150 bp and the fact that the majority of the reads will be off-target, this remains insufficient to adequately resolve mtDNA deletions that occur at frequencies of less than one in a million genomes. Even assuming no off-target reads, the MiSeq® instrument would still only yield about one deletion in ten runs. It is therefore critical that a selection step be performed to limit the number of off-target reads and to enrich for deletion-bearing molecules.

PCR-based methods, including long-distance PCR and real-time quantitative PCR, are among the most frequently employed methods for both selection and amplification of deletions (Cortopassi & Arnheim, 1990; He *et al*., 2002; Chabi *et al*., 2003; Kraytsberg *et al*., 2008). Generally speaking, these assays distinguish wild-type from deleted genomes through exploiting differences in amplicon fragment lengths and amplification efficiencies. Given that they do not select for deleted molecules prior to amplification, one of the main drawbacks is high background signal from contaminating wild-type molecules, thus limiting the effective sensitivity. Furthermore, these bulk PCR assays tend to introduce a number of additional artifacts arising from preferential amplification of small templates (allelic preference), introduction of false deletions through template jumping, and other PCR errors (Kraytsberg & Khrapko, 2005). Real-time quantitative PCR (qPCR) can be quite sensitive, but its reliance on relative differences in crossing thresholds rather than direct quantification makes it more suitable for measuring fold changes rather than absolute deletion frequencies (He *et al*., 2002; Chabi *et al*., 2003). Digital PCR methods, including long single-molecule PCR (long smPCR) (Kraytsberg & Khrapko, 2005; Guo *et al*., 2010) and the random mutation capture assay developed for mtDNA deletions (deletion RMC) (Vermulst *et al*., 2008a,b) achieve direct quantification through the use of single-molecule partitioning in 96-well plates. Partitioning additionally serves to minimize artifacts of template jumping and allelic preference that are common in bulk PCRs (Kraytsberg & Khrapko, 2005). Despite these advantages, this approach becomes laborious and costly when using the wells of a multiwell plate as the partition and yields only a handful of the most common deletions within a sample.

The Digital Deletion Detection (3D) assay shows a marked improvement in specificity, sensitivity, and accuracy over other available methods. This is achieved via a three-step process of selection, amplification, and characterization (i.e., quantification or sequencing). As with deletion RMC, high specificity for deletion-bearing molecules is achieved through the destruction of WT template molecules by restriction endonuclease, thereby selecting for and enriching mutant molecules prior to amplification. Following enrichment, partitioning for digital PCR amplification is performed through the generation of up to 20 000 droplet partitions, the equivalent of over 200 96-well plates, within a single reaction well. Quantification is greatly facilitated through the use of TaqMan reporter probes and cytometry, which allows for rapid enumeration of all partitions that contain an amplifiable template and direct quantification of all deletions within a sample.

One of the unique advancements of the 3D assay is the wealth of single-molecule information that is obtained from cytometric analysis of the droplet partitions. In other mtDNA deletion detection assays, hundreds of wells must be screened to yield a handful of successful amplifications. The corresponding template molecules can only be characterized through the additional steps of gel electrophoresis or sequencing. This process will tend to oversample large clonal deletions and thus may not yield a true representation of the biological diversity of deletions present (see Supplemental Note 3). In contrast, 3D provides an opportunity to robustly screen tens of thousands of droplet partitions, yielding hundreds of positive reactions and allowing analysis of a more complete set of deletions in the sample. Moreover, the demonstrated inverse relationship between template size and the endpoint fluorescent intensity of the droplet partitions (Fig. 3C) can be exploited to reveal information regarding the size and homogeneity of the templates in the sample. By analyzing the amplitude distribution of positive droplets, we were able to accurately predict whether the deletion population consisted of a few clonal expansions or a large collection of random deletions (Figs 3 and S7). In this way, cytometric analysis of the partitions could be used to gather information about the size spectrum of deletion templates in the sample without being subject to the biases inherent in individual cloning or the costs of deep sequencing. We believe that with further development, this relationship could potentially be exploited to open new possibilities for ‘next-generation’ PCR technology that can dynamically sort and collect specific amplification products, similar to fluorescence-activated cell sorting with flow cytometry.

Another advantage of the 3D assay is its ability to adjust the search parameters to measure many different target deletion sets. This is achieved by defining the target deletion space through careful choice of primer locations and the restriction enzyme. This is an important advantage over many existing methods in that random deletions within a target region can be analyzed without knowing the precise breakpoints of the target deletion set *a priori*. It is noteworthy that we were able to measure the deletion loads at both sites simultaneously, given that the minor arc deletion frequency was up to 100-fold less than the major arc. In many other assays, this information would be lost to the dominant signal of the clonal expansions. Importantly, the assay is also neutral with regard to random (i.e., steady-state temporal deletions that occur at low frequency) vs. clonal events (i.e., deletions that have expanded out of the steady-state pool and that occur at relatively high frequency): the assay will detect all deletions that fall within the defined deletion space. Thus, our assay is able to account for gain or loss of steady-state temporal deletions as well as clonal expansions.

Finally, by coupling NGS with the other steps in 3D, we are able to perform high-resolution characterization of millions of breakpoints within a single sequencing run. To demonstrate the utility and sensitivity of this assay, we analyzed deletion loads within the mitochondrial genome of human brain samples. For example, at the ND1/ND2 site, we interrogated over 8 billion mitochondrial genomes and identified over 100 000 genomes with a deletion within our target region. At that site, we were able to characterize 430 individual unique deletions with an average sequencing coverage of 78-fold. Furthermore, based on the specific sequencing coverage, we were able to distinguish between clonally expanded and random, ‘steady-state temporal’ deletions. To our knowledge, no other assay has demonstrated the capability of identifying and analyzing such a large deletion set with comparable resolution.

Digital Deletion analysis allows for unbiased, high-resolution analysis of the full spectrum of deletions within the target site. With this tool, we can better analyze the mechanics and kinetics of deletion acquisition and expansion in aging tissue. Accumulation of mtDNA deletions, particularly in postmitotic tissue, is an important cause of human pathology and aging (Cortopassi & Arnheim, 1990; Meissner *et al*., 2008; Vermulst *et al*., 2008b; Khrapko & Vijg, 2009). While it is known that deletions can accumulate through a process of clonal expansion of a pre-existing pool of deletions, it is unclear whether this or an accelerated rate of *de novo* deletions is the primary driving force behind age-related deletion accumulation (Khrapko, 2011). Previous studies using mathematical simulations of cell division or analysis of the distribution of deletions in tissues conclude that many mtDNA mutations may have an early origin and have been subsequently expanded (Brierley *et al*., 1998; Elson *et al*., 2001; Khrapko *et al*., 2003, 2004; Payne *et al*., 2011). However, work from some of the same groups also leads to the opposite conclusion that mtDNA deletions may be of late origin (Nicholas *et al*., 2009). To address this issue, we used 3D to characterize the absolute deletion frequency and deletion spectrum of aging brain tissue at two regions of the mitochondrial genome. We found that the total deletion load increases, but that the normalized number of unique deletions did not change from younger to older tissue. Furthermore, we observed little change in the size distribution of deletions as well as the relative pools of high- and low-frequency deletions indicating a fairly static spectrum of diversity. An important caveat is that in the present work, we are not actually tracing the dynamics of specific deletions with time, but are rather harvesting snapshots of the deletion burden across several individuals. Thus, we cannot rule out the contribution of newly acquired deletions to later time points. This is particularly true in the case of the common deletion where the dominance of a single-deletion species at this site makes it impossible to determine whether we are observing clonal expansion or rapid re-accumulation of the same deletion. However, at the ND1/ND2 locus, we were able to recover a large diversity of deletions without such site saturation (Figs 6 and S6). Thus, within the time frame analyzed (aged 28–80 years), our data support the hypothesis that expansion (rather than generation of new deletions) dominates the age-related increase in deletion load.

The fact that early mutations are allowed to accumulate to significant levels may be interpreted as evidence for some sort of selective pressure. Precisely what that pressure is, however, remains unclear. Our data show uniform random distribution of deletion lengths at the ND1/ND2 site across all ages. The absence of a shift in the diversity toward accumulation of larger deletions argues against the hypothesis that smaller mtDNA molecules possess a replicative advantage in postmitotic cells (Wallace, 1989; Fukui & Moraes, 2009). Our data are not inconsistent with *in silico* experiments that predict that clonal expansion can result from random genetic drift without the aid of selection (Coller *et al*., 2001; Elson *et al*., 2001). While this model has been somewhat validated for point mutations (Durham *et al*., 2006), other selective mechanisms for deletions cannot be ruled out (de Grey, 2009). 3D will allow us to perform longitudinal studies that can trace the kinetics of clonal expansion of real deletions which will allow us to better test the *in silico* models with data from living cells.

The 3D/NGS data demonstrate that we now have the technology to perform high-resolution analysis and detailed characterization of extremely rare deletion events. Importantly, it also provides the means to begin to use mtDNA deletions as biomarkers for disease. Although mtDNA deletions accumulate readily in skeletal muscle and brain tissue, they exist at extremely low levels in blood and other rapidly proliferating tissue (DiMauro & Hirano, 1993). This has been a great hindrance to the development of blood-based biomarker assays that could be used for noninvasive screening and early detection of mitochondrial deletion diseases. Digital Deletion Detection provides an important new tool that will allow researchers to better study the mechanisms of deletion formation, their mechanisms of expansion, and their role in the etiology of aging and disease.

## Experimental procedures

### Human brain tissue

Human histologically normal brain obtained from informed patients was obtained from the tissue depository of the Department of Neurological Surgery at the University of Washington. Tissue and demographic information was obtained in accord with an IRB-approved protocol (Table 1).

### DNA isolation

To obtain whole DNA from human brain tissue, tissue samples (50–250 mg) were immersed in 5 mL homogenization medium (0.32 m sucrose, 1 mm EDTA, 10 mm Tris–HCl, pH 7.8) and disrupted with a glass Dounce-type homogenizer. The homogenate was transferred to a 15-mL tube and centrifuged at 4000 g. The pellet was resuspended in 3 mL lysis buffer (10 mm Tris–HCl, pH 8.0, 150 mm NaCl, 20 mm EDTA, 1% SDS, and 0.2 mg mL^−1^ proteinase K) and incubated at 55 °C for 3 h. DNA was isolated by phenol–chloroform extraction followed by isopropanol precipitation.

### Endonucleolytic enrichment of mtDNA deletions

Rare deletion-bearing molecules were selectively enriched through endonucleolytic destruction of wild-type target sites. First, a 400 μL digestion reaction mixture was prepared containing 10 μg of genomic DNA, 8 μL (800 U) of *TaqI* (New England Biolabs, Ipswich, MA, USA), and *TaqI* reaction buffer (Fermentas, Vilnius, Lithuania). The reaction mixture was divided into 4 × 100 μL reactions and incubated at 65 °C for 4–6 h. An additional 200 U of *TaqI* was added to each reaction every hour. After each *TaqI* addition, samples were thoroughly mixed and briefly centrifuged to ensure efficient digestion. Following the digestion procedure, the reactions were recombined, extracted once with phenol/chloroform/isoamyl alcohol (25:24:1, v/v), precipitated by ethanol, and resuspended in 1 mm Tris, pH 8.

### TaqMan probe and primer design

The following primer/probe sets were used with human total DNA for mtDNA deletion detection. Control site: 5′-CTA AAA ATA TTA AAC ACA AAC TAC CAC CTA CCTC-3′ (forward primer), 5′-GTT CAT TTT GGT TCT CAG GGT TTG TTA TAA-3′ (reverse primer), and 5′-6FAM- CCT CAC CAA AGC CCA TA-MGB-3′ (probe). ND1/ND2 site: 5′-CGC CAC ATC TAC CAT CACC-3′ (forward primer), 5′-GAT TAT GGA TGC GGT TGC TT-3′ (reverse primer), and 5′-6FAM-TTG ATG GCA GCT TCT GT-MGB-3′ (probe). Common deletion site: 5′-TAC CCC CTC TAG AGC CCA CT-3′ (forward primer), 5′-GAG GAA AGG TAT TCC TGC TAA TGCT-3′ (reverse primer), and 5′-6FAM-TGG CCC ACC ATA ATT-MGB-3′ (probe).

### Droplet digital PCR

The final concentration of digested DNA was adjusted to yield less than ~3500 positive molecules per μL, which is within the range of linearity for the Poisson calculation (Pinheiro *et al*., 2012). Reaction mixtures (25 μL) contained ddPCR Master Mix (Bio-Rad, Hercules, CA, USA), 250 nm TaqMan probe, and 1–2 μL of digested DNA (0–2 μg total). Appropriate flanking primers were added at either 900 nm or 45 nm for the quantification and sequencing process pathways, respectively (see Supplementary Notes 1 and 2). Reaction droplets were made by applying 20 μL of each reaction mixture to a droplet generator DG8 cartridge (Bio-Rad) for use in the QX100 Droplet Generator (Bio-Rad). Following droplet generation, 38 μL of the droplet emulsion was carefully transferred to a Twin.tec semi-skirted 96-well PCR plate (Eppendorf, Hamburg, Germany), which was then heat-sealed with a pierceable foil sheet. To amplify the fragments, thermal cycling was carried out using the following protocol: initial denaturation step at 95 °C for 10 min, followed by 40 cycles of 94 °C for 30 s, and 63.5 °C for 4 min. The thermally cycled droplets were either (i) analyzed by flow cytometry for fluorescence analysis and quantification of deletion frequencies (see Methods SI) or (ii) disrupted and the PCR products recovered and sequenced in order to verify deletions and characterize the deletion sites (see Methods SI). All experiments were performed in triplicate.

### Analysis of fluorescence amplitude and quantification of deletions

Following normal thermal cycling, droplets were individually scanned using the QX100™ Droplet Digital™ PCR system (Bio-Rad). Positive (deletion-bearing) and negative droplets were distinguished on the basis of fluorescence amplitude using a global threshold. The number of mutant genomes per droplet was calculated automatically by the accompanying software (QuantaSoft, Bio-Rad) using Poisson statistics as described elsewhere (Hindson *et al*., 2011). Quantification of deletion frequency requires ddPCR amplification using two primer sets. The first primer set flanks the test region and measures the concentration of deletion-bearing molecules. The second primer set flanks a distant region in the genome that bears no restriction recognition sites. This second or control set measures the concentration of all mtDNA genomes. Because *de novo* deletions are so rare, reactions using the different primer sets must be run using different dilutions of the digested DNA, and the results normalized against the mass of total DNA in the reaction. Deletion frequency is calculated by taking the ratio of the normalized concentrations of deletion-bearing mtDNA molecules to the total mtDNA molecules screened. Reactions that yielded < 10 positive droplets per well were scored conservatively as having no positives above background (Pinheiro *et al*., 2012).

### Library preparation and Illumina sequencing

Human ND1/ND2 ddPCR amplification products were subjected to template conversion as described in Methods SI. Reactions were cleaned using the ZR-96 Clean and concentrator-5 kit (Zymo Research, Irvine, CA, USA). Template concentrations were calculated using the Quant-iT™ PicoGreen dsDNA Assay Kit (Invitrogen, Carlsbad, CA, USA) following manufacturer’s recommended protocol. Samples were then diluted to 0.2 ng μL^−1^ in 10 mm Tris, pH 8.0, 1 mm EDTA (TE). Fragmentation, adaptor ligation, and index ligation were accomplished using the Nextera XT DNA Sample Preparation Kit (Illumina) following the recommended protocol.

Because the common deletion breakpoint is within 100 bp of the 3′ end of the amplicon, the normal tagmentation protocol could not be followed. Instead, adaptors were added directly via PCR using the following primers: 5′-TCG TCG GCA GCG TCA GAT GTG TAT AAG AGA CAG NNN NCG TAT GGC CCA CCA TAA TTA CC (forward) and 5′-GTC TCG TGG GCT CGG AGA TGT GTA TAA GAG ACA GNN NNG AGG AAA GGT ATT CCT GCT AAT GCT-3′ (reverse). Thermal cycling consisted of an initial denaturation at 95 °C for 10 min, followed by 8 cycles of 94 °C for 30 s, 58 °C for 30 s, and 63.5 °C for 4 min. Reactions were cleaned using the ZR-96 Clean and concentrator-5 kit (Zymo Research) at concentrations and dilutions performed above. 5 μL of 0.2 ng μL^−1^ DNA was mixed with 20 μL TD buffer prior to PCR amplification in the Nextera XT DNE Sample Prep workflow. The rest of the Nextera XT protocol was performed according to recommended procedures. Indexed ND1/ND2 and common deletion fragments were pooled for all patients and sequenced using the MiSeq Personal Sequencing System (Illumina) (see Methods SI). FASTQ files for each patient were deposited in the NCBI Sequence Read Archive (SRA) under project accession number SRP027401.

### Reconstruction experiments

Genomic DNA was isolated from HCT 116 cells, chosen for its relatively low endogenous deletion frequency of 1.8 × 10^−7^. Following *TaqI* digestion, a series of 10-fold serial dilutions of the genomic DNA were prepared, ranging over eight orders of magnitude. A 997-bp deletion was isolated, amplified, and cloned into a vector for use as a control molecule (Fig. 5B). Approximately 600 ng of the 3534Δ997 control plasmid was serially diluted 100 million fold and subjected to a preliminary 3D analysis to calculate the absolute concentration of molecules within the dilution. To each of the genomic dilutions, three copies of the 3534Δ997 control plasmid were added per microliter of reaction. The reaction mixtures were then partitioned, cycled, and the droplets analyzed to determine whether the small concentration of the control molecules could be accurately assessed even in the presence of high concentrations of background, HCT 116 DNA.

### Heterogeneous population reconstruction experiments

Three control plasmids (3534Δ997, 3719Δ809, and 3871Δ492) were isolated from *POLG*^D274A^ HeLa cells as described above (see also Fig. 3). Each plasmid was serially diluted and subjected to preliminary 3D analysis in order to calculate the concentration of molecules within each dilution. Based on these quantifications, 300 molecules μL^−1^ per template were subjected to another round of 3D analysis, either separately or combined into a single reaction.

### Regression analysis

Linear regression analyses were performed in R using the built-in Stats package (R Core Team, 2013). Significance of linear models was calculated using the *F*-test against the null hypothesis of no correlation between the variables tested.

## Supporting Information

**Fig. S1.** Effect of primer concentration on amplified copy number. **Fig. S2.** Relative recovery proportions at various stages of 3D analysis. **Fig. S3.** 3D analysis of mtDNA isolated from PolgWT and PolgD257A knock-in mice. **Fig. S4.** Analysis of mitochondrial DNA copy number from human brain tissue samples. **Fig. S5.** Analysis of ddPCR droplet counts from sequenced patient samples. **Fig. S6.** Deletion sites are not fully saturated. **Fig. S7.** Distribution of positive droplets from ddPCR of human brain. **Fig. S8.** High resolution analysis of deletion dynamics.

**Data S1.** Supplementary notes, methods and figures. **Data S1A.** Optimization of amplification factors. **Data S1B.** Residual amplification bias. **Data S1C.** Biases inherent in downstream applications. **Data S1D.** Validation in biological samples. **Data S1E.** Validation of sampling and analysis. **Data S1F**. Disruption of droplet emulsions **Data S2.** Tables of all accepted deletions for each patient. Sequence data deposited in the NCBI Sequence Read Archive under project accession number SRP027401.
