# Supplementary material for: Targeted enrichment and high-resolution digital profiling of mitochondrial DNA deletions in human brain
Source: Aging Cell. 2013 Sep 11;13(1):29–38. doi: 10.1111/acel.12146 (PMC4068027; doi:10.1111/acel.12146)
Supplement: Supplementary file 1 — Fig. S1. Effect of primer concentration on amplified copy number. Fig. S2. Relative recovery proportions at various stages of 3D analysis. Fig. S3. 3D analysis of mtDNA isolated from PolgWT and PolgD257A knock-in mice. Fig. S4. Analysis of mitochondrial DNA copy number from human brain tissue samples. Fig. S5. Analysis of ddPCR droplet counts from sequenced patient samples. Fig. S6. Deletion sites are not fully saturated. Fig. S7. Distribution of positive droplets from ddPCR of human brain. Fig. S8. High resolution analysis of deletion dynamics. [file acel0013-0029-sd1.pdf]

High-Resolution Digital Analysis of Random Mitochondrial DNA Deletions

Sean D. Taylor, Nolan G. Ericson, Joshua N. Burton, Tomas A. Prolla, John R. Silber, Jay Shendure, Jason H. Bielas

Supplementary Notes

Supplementary Note 1: Optimization of amplification factors

Although 3D is able to accurately quantify the total number of heterogeneous deletions from a population, certain challenges remain for characterization of the unique events from such a population. For example, in any PCR-based reaction it is generally likely that smaller templates will be preferentially amplified over larger ones given that smaller quantities of dNTPs are consumed and smaller quantities of inhibiting pyrophosphates are produced per cycle. This is especially problematic in bulk PCR reactions where the smaller template will out-compete larger templates for amplification. Such asymmetric amplification may have a significant impact on downstream characterization procedures such as analytical gel electrophoresis, cloning, and sequencing. The hyper-partitioning of the 3D reaction largely circumvents this problem by providing separate reaction vessels for each template, thus preventing direct competition between different sized templates. Nonetheless, within each reaction droplet, smaller templates appear to produce more amplified fragments than an equivalent droplet bearing a larger template as evidenced by the greater average droplet fluorescence amplitude for shorter templates (Figure 3C). This hypothesis was supported by quantitative analysis of amplification products using capillary gel electrophoresis (Figure S1A)

We reasoned that if the primer concentration were significantly reduced, then the limited primer pool would set an upper limit on the amplification across all template sizes. In this way the amplification bias might be minimized and the amplified copy number normalized. We therefore repeated the 3D analysis, reducing the primer concentration 20-fold from 900 nM to 45 nM. Under conditions of limiting primer, the amplitudes were reduced nearly 2-fold, resulting in a smaller amplification bias across the different template sizes (Figure S1B). This result was confirmed by directly measuring the concentration of amplified fragments via capillary gel electrophoresis (Figure S1A). Importantly, we found that although the endpoint amplitude was reduced, the quantification of absolute plasmid concentrations by 3D remained unaffected (Figure S1C). Because the quantification did not appear to be affected by the

primer concentration, we used two primer concentrations in all subsequent reactions. The higher primer concentration was used for quantification and allowed us to assess deletion heterogeneity. The lower primer concentration was used to normalize amplicon copy number and was used for cloning and sequencing analysis.

Supplementary Note 2: Residual amplification bias

We were able to substantially reduce the amplification bias in 3D by reducing the primer concentration such that it becomes the limiting reagent. By doing so, we set an upper limit on the total number of amplicons that can be generated from any given template. While the bias was greatly reduced, it was not altogether eliminated (Figure 3). This is because template compartmentalization obeys a Poisson distribution and a small subset of reaction droplets will contain more than one template. In such cases, amplification of the smaller template will be favored as in conventional PCR reactions. If the input concentrations of each template are known (as is the case for our control reactions), then a statistical correction can be applied using combinatorics and the Poisson equation in order to account for biased amplification in partitions with multiple templates.

If we assume that in cases where multiple templates are found within the same partition that only the shortest template (T_1) will be amplified, then we must first calculate the total number of droplets (N_1) that contain at least one molecule of T_1:

$$N_1 = N_{Total} \times \sum_{k=1}^{n} \left[Poiss(\lambda, k) \times \sum_{r=1}^{k} \left[\binom{k}{k-r} \times P_1^k \times (1-P_1)^{k-r} \right] \right] \tag{1}$$

Where N_{Total} is the total number of droplets, P_1 is the probability of selecting the short template once from a pool of all available templates (equal to the relative concentration of the template), and *n* is the maximum number of templates found inside a single droplet. The average number of templates per droplet (λ) is found according to equation 3:

$$\lambda = \frac{M * V_d}{1000} \tag{2}$$

where *M* is the total concentration of templates per µl, and V_d is the mean droplet volume (0.91 nl)(Pinheiro *et al.* 2012). The Poisson function used in (1) is expressed as

$$Poiss(\lambda, k) = \frac{\lambda^k e^{-\lambda}}{k!} \tag{3}$$

Next we calculate the number of droplets (N_2) that contain at least one copy of the next shortest template (T_2), excluding those that contain any longer piece:

$$N_2 = N_{Total} \times \sum_{k=1}^{n} \left[Poiss(\lambda, k) \times \sum_{r=1}^{k} \left[\binom{k}{k-r} \times P_2^{\,r} \times (1 - P_1 - P_2)^{k-r} \right] \right] \quad (4)$$

This process is repeated until all templates have been accounted for. Comparison of these values provides a correction for the relative amplification factor for each template. We applied this procedure to the relative amplification factors obtained from capillary gel electrophoresis, using, N_{Total}=45,475, λ=0.80171, *n*=6, and the following template concentrations (*P*):

Template	*P*	*N*	Poisson Correction		Relative amplification factor		Template Proportion
3534Δ997	0.3449	10985	0.56	*x*	1.9	=	1.1
3719Δ809	0.3286	7988	0.76	*x*	1.2	=	0.9
3871Δ492	0.3265	6103	1.00	*x*	1.0	=	1.0

As the total concentration of positive molecules is reduced in the sample, so is the need for this statistical correction.

Supplementary Note 3: Biases inherent in downstream applications

By reducing the primer concentration, amplification is normalized across targets of all sizes such that the molar ratios of the amplified products are proportional to the ratios of their respective templates. An important caveat however, is that many downstream applications used to characterize the deletions also present their own biases (Figure S2). For example, cloning of the PCR products typically favors ligation of small inserts into the vector (corresponding to larger deletions). This problem is largely ameliorated by employing deep sequencing technology which bypasses the ligation and transformation steps. This also offers the advantage of being a far more comprehensive characterization than could be obtained by selecting some subset of colonies for analysis.

Supplementary Note 4: Validation in biological samples

Homozygous knock-in mice expressing exonuclease-deficient (exo$^-$) *Polg* demonstrate a significant a in deletion frequency (Vermulst *et al.* 2008; Edgar *et al.* 2009). In order to validate that 3D can be used to measure deletions from biological samples, we applied 3D to mtDNA isolated from muscle samples of homozygous mice that contained either the WT *Polg* allele or an exo$^-$ variant harboring a D257A mutation in the second exonuclease proof-reading domain (*Polg*^{D257A}) (Vermulst *et al.* 2007; Vermulst *et al.* 2008). At one site within the major arc of the genome, we measured a deletion frequency of 7 deletions per 10^7 genomes for the WT mouse (i.e. 7 x 10^{-7}) and 5.7 x 10^{-6} for the mutant mouse (Figure S3). The sensitivity of the assay is further highlighted at a second site encompassing the light chain origin of replication, where the deletion frequency was found to increase from 1 x 10^{-8} to 1 x 10^{-6} (Figure S3). These increases in deletion frequency are in agreement with previous estimates for *Polg*^{D257A}-induced deletion loads(Vermulst *et al.* 2008). With the use of 3D, we are able to additionally determine the absolute deletion frequency at these sites.

Supplementary Note 5: Validation of sampling and analysis

There are several steps in the analysis where over-/under-sampling of genomes or analysis bottlenecks may lead to artifacts that could potentially skew the data and/or influence data interpretation. In order to guard against any such factors we performed the following additional analyses .

First we examined the number of genomes isolated from each patient (Figure S4). Similar amounts of mtDNA were obtained from each patient sample. We also verified that the mtDNA/nuclear copy number ratio was constant across all patients (data not shown). Together, these data demonstrate that mtDNA was obtained from approximately the same number of cells. We also verified that similar amounts of mtDNA were used as inputs in the deletion screen (Figure S4). Given that the mutation frequency is found by normalizing the concentration of deletion bearing genomes to the total number of genomes screened, the input concentrations need not be strictly uniform. We found that all input concentrations were similar for each assay performed. Further, there is no correlation between input concentration and deletion frequency. Thus, no bias has arisen from over- or under-sampling of tissue mass or of the mitochondrial genomes of the various patients.

Given that the number of deletions profiled through sequencing analysis depends directly on the amount of input DNA received from the ddPCR step, we next examined the number of droplets

generated during the ddPCR step for each of the sequenced samples. Droplet counts are uniform across all patients, with an average droplet count of approximately 14,000. (Figure S5A). Additionally, each assay should contain at least ten positive droplets in order to achieve an appropriate signal to noise ratio (Pinheiro *et al.* 2012). At the common deletion site, the average positive droplet count is 3661, with a minimum of 455 (Figure S5B). At the ND1/ND2 site, the average positive droplet count is 474, with a minimum value of 77 (Figure S5B). Analysis of these droplet counts demonstrates that each patient is adequately sampled above the sensitivity threshold and without cutting off the distribution tails of the deletion sets.

As an additional check to ensure that the number of deletions obtained from NGS was supported by the ddPCR data, we also compared the number of accepted unique deletions for each sampled patient against the positive droplet count. Assuming that each droplet contains a single template, the number of unique deletions reported should not exceed the number of positive droplets. In all cases, we see that this is indeed the case, demonstrating that the reported numbers of unique deletions are reasonable given the number of positive droplets observed (Figure S5B).

We also tested the possibility that the diversity for deletions may have reached saturation at each mutational target site (i.e. all biologically possible deletions in the target site are accounted for in the sample). If this were the case, it would potentially mask our ability to detect further breakpoint diversification. To analyze this, we first pooled all the observed deletions across all sampled patients. In this combined set, we observed a total of 430 distinct deletion classes at the ND1/ND2 site, with 21 at the common deletion site (Figure S6). If the diversity within a patient has reached saturation, the number of unique deletions in the patient should approach that observed in the pooled set. The degree of saturation at each site can thus be found by taking the ratio of patient specific deletions against the unique deletions found in the pooled set (Figure S6). This ratio was also found to be uncorrelated with age. This analysis shows that the median saturation was 13.2% and 23.8% for the ND1/ND2 and common deletion sites respectively, demonstrating a capacity for further diversification in all sampled patients. It is interesting to note that while the canonical common deletion still accounts for over 99% of the deletions actually observed (Figure 6), this analysis only shows about 23% saturation of possible deletions at the common deletion site. This indicates that while the canonical deletion may be mechanistically favored (Krishnan *et al.* 2008), other deletions are certainly possible at this site. Expansion of this analysis may be informative as to the relative rates of formation of the different deletion classes at this site and may lead to a better mechanistic understanding of deletion formation in

the mitochondria. The analysis demonstrates a high degree of diversity across patients, indicating that we have not reached saturation in our measurements of unique deletion loads.

Finally, the total number of unique deletions was normalized against the number of deletion bearing molecules screened (Figure 5B). This was done in order to account for differences in the concentration of deletion bearing molecules as input into the sequencing analysis, For example, if a lower concentration of input molecules is used for sequencing analysis, we would expect to detect fewer unique events. By normalizing against the number of deletion molecules screened, we are able to obtain a more accurate comparison of diversity across patients. However, the results and conclusions remain unchanged, namely that there is no significant increase in diversity with age.

Taken together we feel confident that we have taken into account all potential variations in sampling, potential bottlenecks, and processing artifacts, and we are confident that there are no hidden variables that might erroneously lead the conclusion of a constant diversity of deletion types

Supplementary Methods

Disruption of droplet emulsions

Following thermal cycling, droplets were re-suspended by adding 1 volume of droplet reader oil and gently pipetting up and down, after which the emulsion was transferred to a clean 1.7 ml microcentrifuge tube. The volume of the solution was adjusted to 200 µl, either through addition or removal of excess droplet reader oil. Emulsions were disrupted by addition of 200 µl chloroform and extracted twice with 150 µl 1mM Tris, pH 8, following which the aqueous phases were removed to a fresh tube. The combined aqueous phases were then extracted again with phenol/chloroform/isoamyl alcohol (25:24:1, v/v) and the DNA precipitated with ethanol. The precipitated DNA was washed once with 70% ethanol, air-dried for 5 min, and re-suspended in 10–20 µl water.

Template conversion

The ddPCR Master Mix used in the initial amplification contains dUTP in place of dTTP, making it unsuitable for Illumina amplification chemistry. A second round of PCR is needed to generate thymidine bearing amplicons. Additionally, nonspecific products often amplify in the droplets. Although these artifacts do not bind the TaqMan probe and therefore do not affect quantification, they do interfere with cloning and sequencing of true deletion products. In order to select target-specific amplicons, the

second round of PCR uses a nested primer that anneals to the TaqMan probe site paired with the initial reverse primer. Reaction mixtures (25 µl) for the nested PCR contained GoTaq® Hot Start Colorless Master Mix, 900 nM each primer, and 5 µl of the resuspended DNA from the initial ddPCR. Thermal cycling consisted of initial denaturation at 95 °C for 10 min, followed by 8 cycles of 94 °C for 30 sec, 58 °C for 30 sec and 63.5 °C for 4 min.

Illumina sequencing and analysis of sequencing results

Indexed ND1/ND2 and common deletion fragments were pooled for all patients and then mixed with an equal volume of denatured 12.5 pM PhiX control DNA. The library was then loaded onto the MiSeq Personal Sequencing System (Illumina) using 150 nucleotides of paired-end analysis. At the completion of the sequencing run, demultiplexed Fastq files for each sample were automatically generated. To call deletions in the mitochondrial genomes, the sequence reads were aligned to the reference human mitochondrial genome. A deletion in the subject was indicated by "split read" alignments in which part of a read aligns to one part of the mitochondrial genome, and the rest of the read aligns to another part. Hits that did not show evidence of the exact same deletion in both reads of a read pair were discarded, as were hits that resulted in coverage gaps in the query sequence. Point mutations caused by sequencing errors can lead to slight misalignment of deletion breakpoints. This was observable by the presence of 'satellite' deletions, deletions with low read numbers with breakpoints and lengths similar to those of 'parental' deletions supported by large numbers of reads. In order to filter erroneous breakpoint calls, any two deletions with breakpoints within a specified distance of each other (13 bp for the common deletion site, 5 bp for the ND1/ND2 deletion site) were considered to be the same. The most commonly appearing deletion in each cluster was taken as the "correct" one. Furthermore, in order to be reported as a true deletion event, each deletion was required to be supported by at least 10 read pairs.

***Polg* knock-in mice**

Hind limb skeletal muscle samples from three homozygous *Polg* ^{WT} and three *Polg*^{D257} knock-in mice, age 13-15 months, were obtained as published previously (Kujoth *et al.* 2005). Mouse mitochondrial DNA was isolated and purified as described in the main text.

TaqMan probe and primer design for mouse experiments

The following primer/probe sets were used with mouse total DNA for mtDNA deletion detection. Control site: 5'- GAC ACA AAC TAA AAA GCT CA-3' (forward primer), 5'- TAA GTG TCC TGC AGT AAT GT-

3’ (reverse primer), 5’- 6FAM- CCA ATG GCA TTA GCA GTC CGG C-MGB-3’ (probe). Major arc: 5’- AGG CCA CCA CAC TCC TAT TG-3’ (forward primer), 5’- AAT GCT AGG CGT TTG ATT GG-3’ (reverse primer), 5’-6FAM-AAG GAC TAC GAT ATG GTA TAA-MGB-3’ (probe1), 5’-6FAM-TGA GGT CTG GGT CATT-MGB-3’ (probe2) . O_L site: 5’- CAA TAA CCC TAC CCC TAG CC-3’ (forward), 5’- GTC AGT TTC CAA AGC CTC CA-3’ (reverse), 5’-6FAM- ACT AGT ATA TCC TAA ACT TC-MGB-3’ (probe1), 5’-6FAM- TGC TTT TGT TAT AAT TTT C-MGB-3’ (probe2).

Capillary gel electrophoresis and densitometry

Capillary gel electrophoresis and densitometry were performed using the QIAxcel Advanced Gel Electrophoresis System (Qiagen). Aliquots of the re-suspended DNA (1–5 µl) were removed and diluted for use in the capillary system following manufacturer’s recommended protocols.

Supplementary Figures

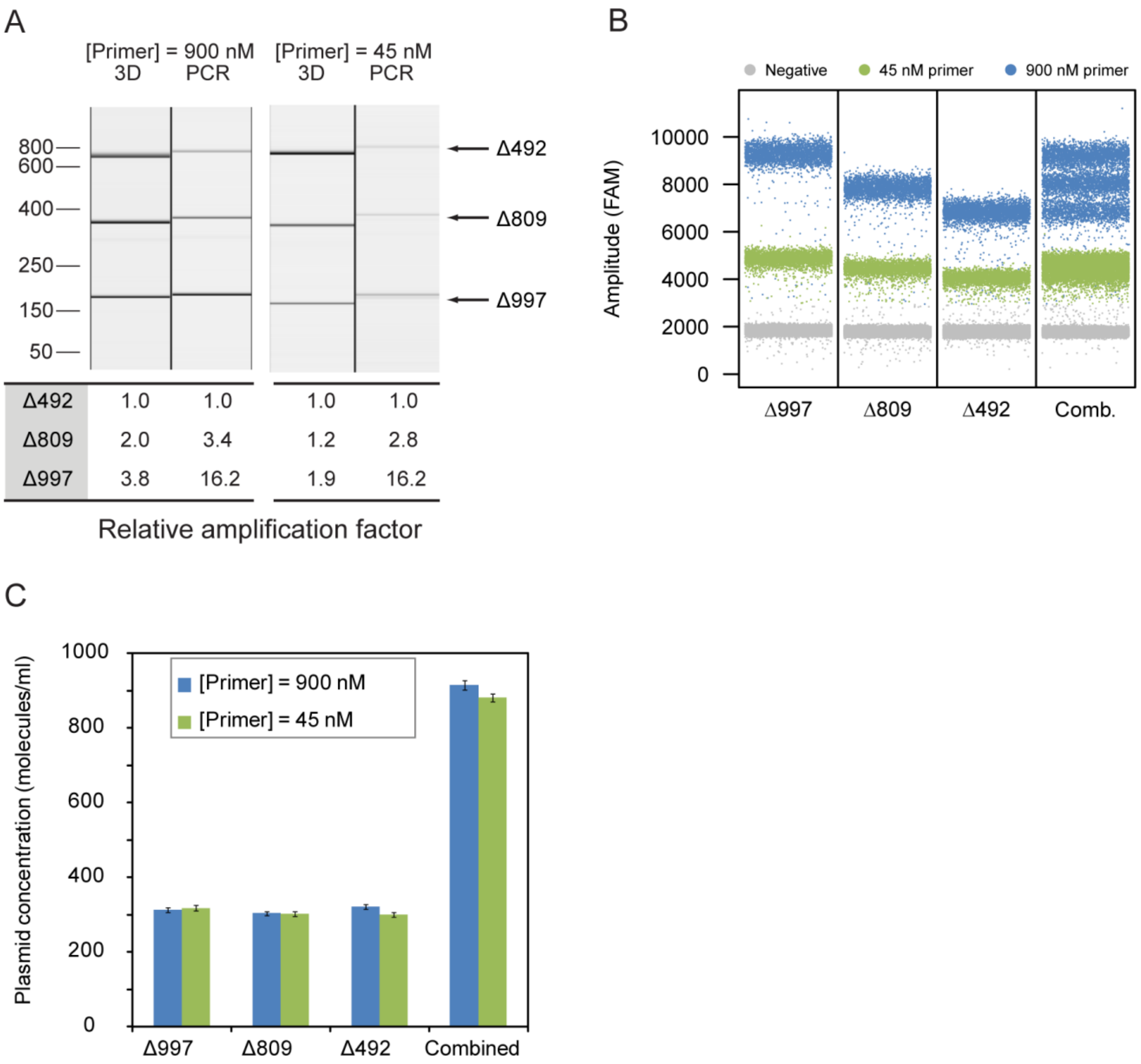

Figure S1. Effect of primer concentration on amplified copy number. (A) Analysis of relative concentration of amplicons using capillary gel electrophoresis. 3D and conventional amplification was performed using two primer concentrations. The droplets were disrupted and the products were resolved and quantified via densitometry. Concentrations were normalized against the product length and compared against the Δ492 product in each reaction in order to calculate the relative amplification factor for each band. (B) Droplet plot showing the effect of primer concentration on relative fluorescence amplitudes. (C) Measured deletion concentration for individual and combined templates at both primer concentrations. Error bars indicate the Poisson 95% confidence intervals for each concentration determination.

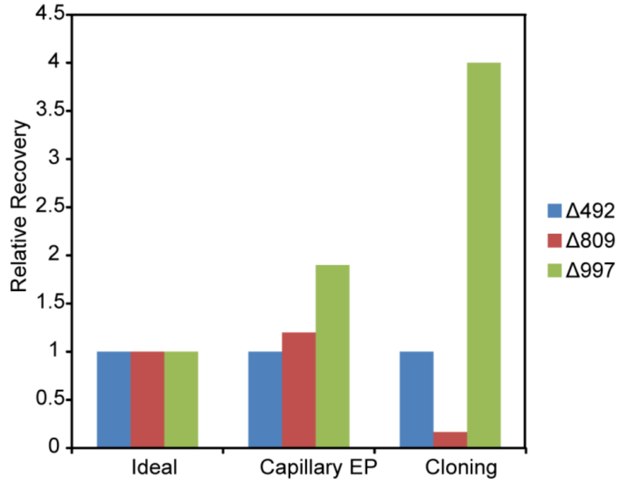

Figure S2. Relative recovery proportions at various stages of 3D analysis. Values are normalized against Δ492. Control plasmids were mixed at equal proportions in the initial ddPCR amplification reaction (Ideal). Droplets were disrupted and the proportion of individual amplicons measured by capillary electrophoresis as described in Figure S1A (Capillary EP). In the few cases where multiple templates are incorporated within the same droplet, the shorter template is preferentially amplified. Amplicons were also cloned and individual bacterial colonies analyzed to determine the proportion of fragments recovered. Cloning yields erratic proportionate recovery but clearly favors shorter templates.

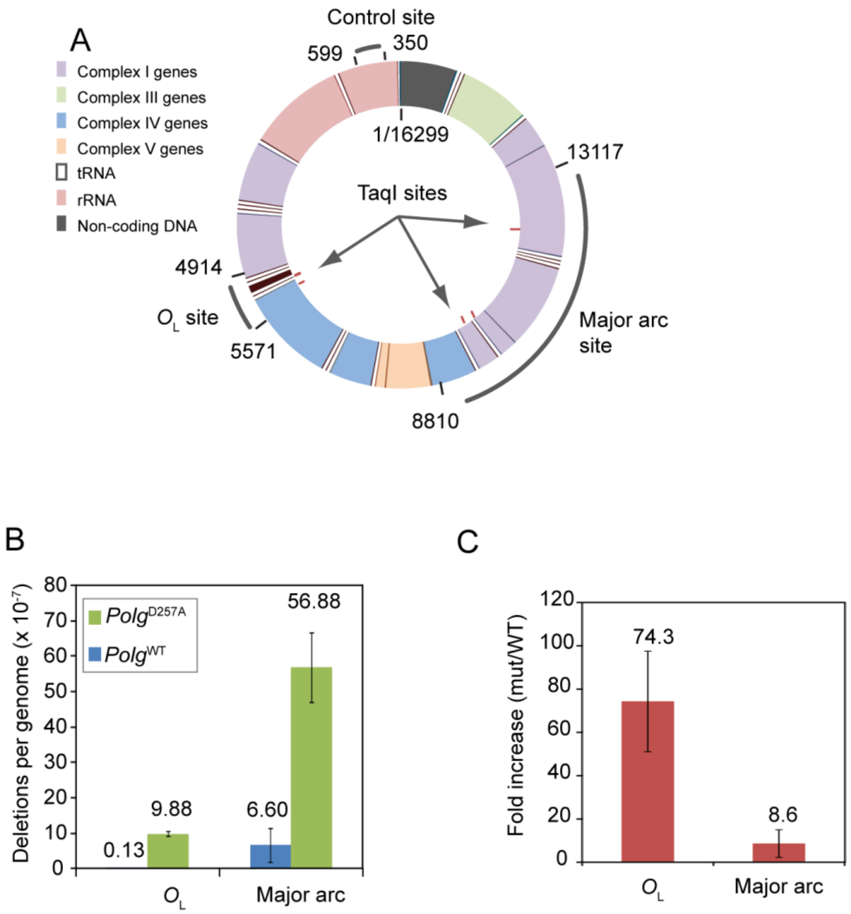

Figure S3. 3D analysis of mtDNA isolated from PolgWT and PolgD257A knock-in mice. (A) Primer locations for the major arc and O_L sites of the mouse mitochondrial genome are denoted. Each primer set flanks three *TaqI* restriction sites. The control site is located in the 12S rRNA gene. **(B)** Quantification of absolute mtDNA deletion frequency (± s.e.m.) at each site for *Polg*^{WT} and *Polg*^{D257A} mice. **(C)** Fold increase in mtDNA deletion frequency for *Polg*^{D257A} mice over wild type at each site.

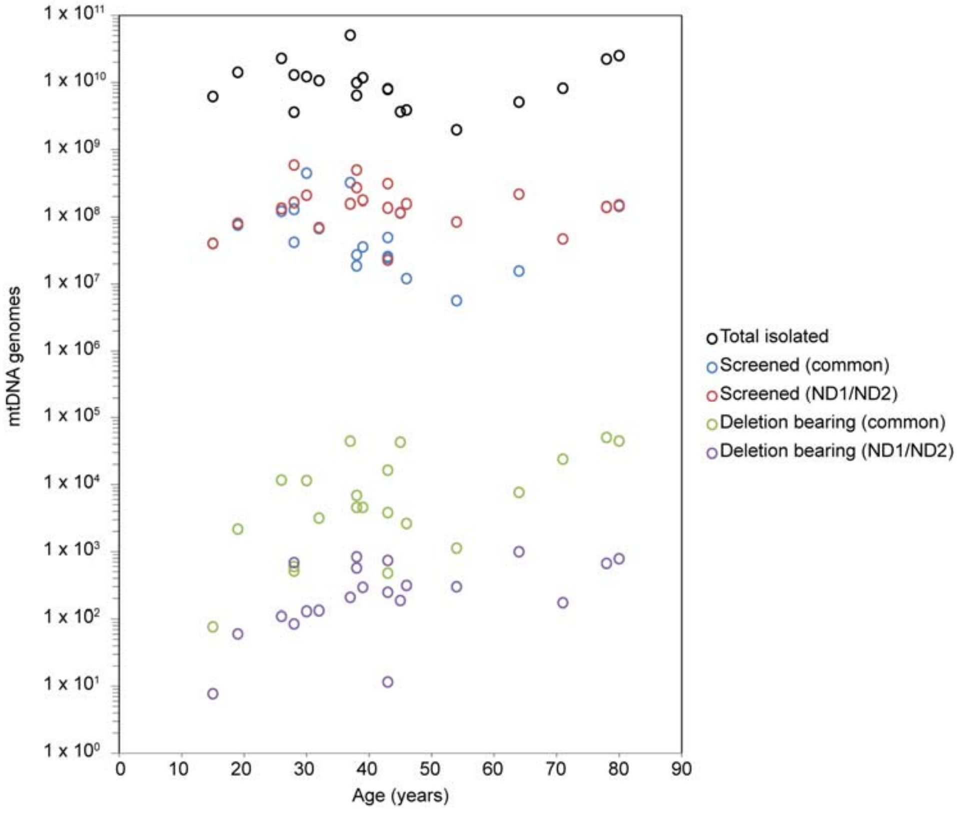

Figure S4. Analysis of mitochondrial DNA copy number from human brain tissue samples. The total number of mitochondrial genomes isolated from each tissue sample is plotted against the patient age. Also shown is the total number genomes screened for each patient in the common and ND1/ND2 deletion assays, as well as the number of deletion bearing genomes detected for each patient.

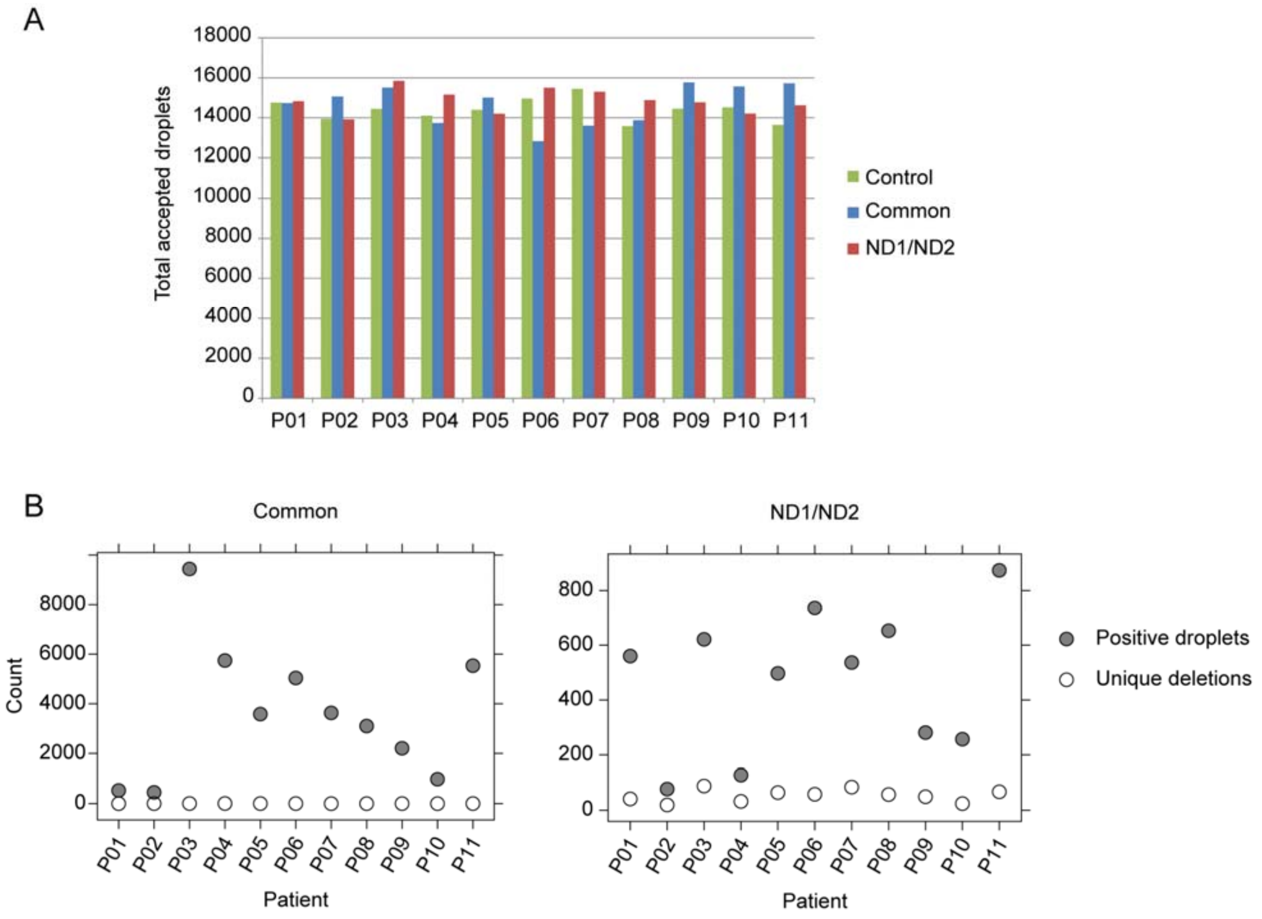

Figure S5. Analysis of ddPCR droplet counts from sequenced patient samples. (A) Total accepted droplet counts is shown for the subset of patients that were used for NGS deletion profiling. Droplet counts are shown for each primer set (control site, common deletion site, and ND1/ND2 site). All droplet counts are above the 10,000 minimum count threshold. **(B)** Positive droplet counts (shaded circles) for the common and ND1/ND2 deletion sites. A minimum of 10 positive droplets are needed to ensure detection and amplification above the sensitivity threshold. The total number of accepted unique deletions from NGS analysis is also shown for each patient (empty circles). Assuming each droplet contains a single deletion bearing template, the number of unique deletions should not exceed the positive droplet count.

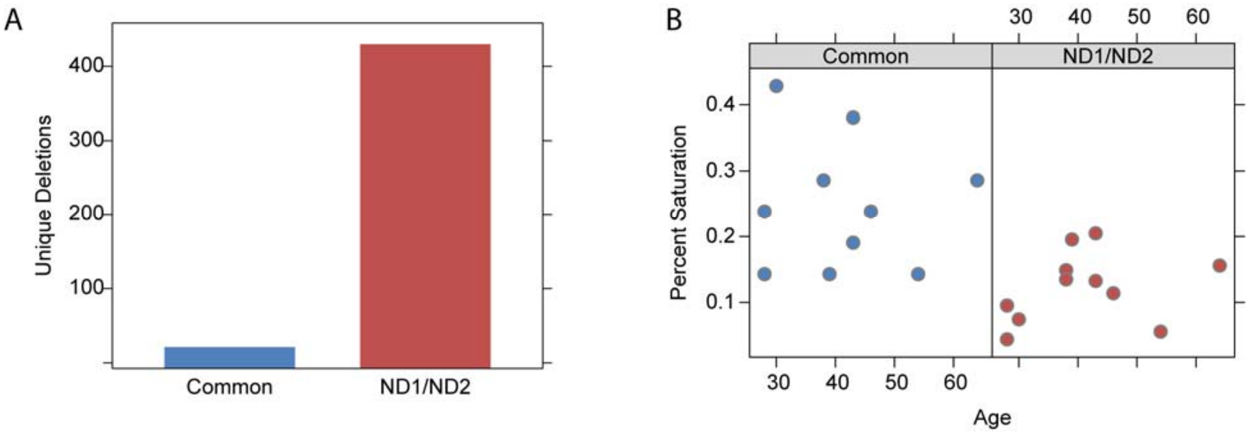

Figure S6. Deletion sites are not fully saturated. (A) The cumulative total of unique deletions pooled across the subset of sequenced patients at the Common (21) and ND1/ND2 (430) site. (B) The fraction of the pooled total of distinct deletions present in each patient comprises the saturation of the deletion site. The common deletion showed a median saturation of 23.8%, interquartile range 16.6% - 28.5%. The ND1/ND2 site showed a median saturation of 13.2%, interquartile range 8.4% - 15.2%. Degree of saturation showed no correlation with age at either site.

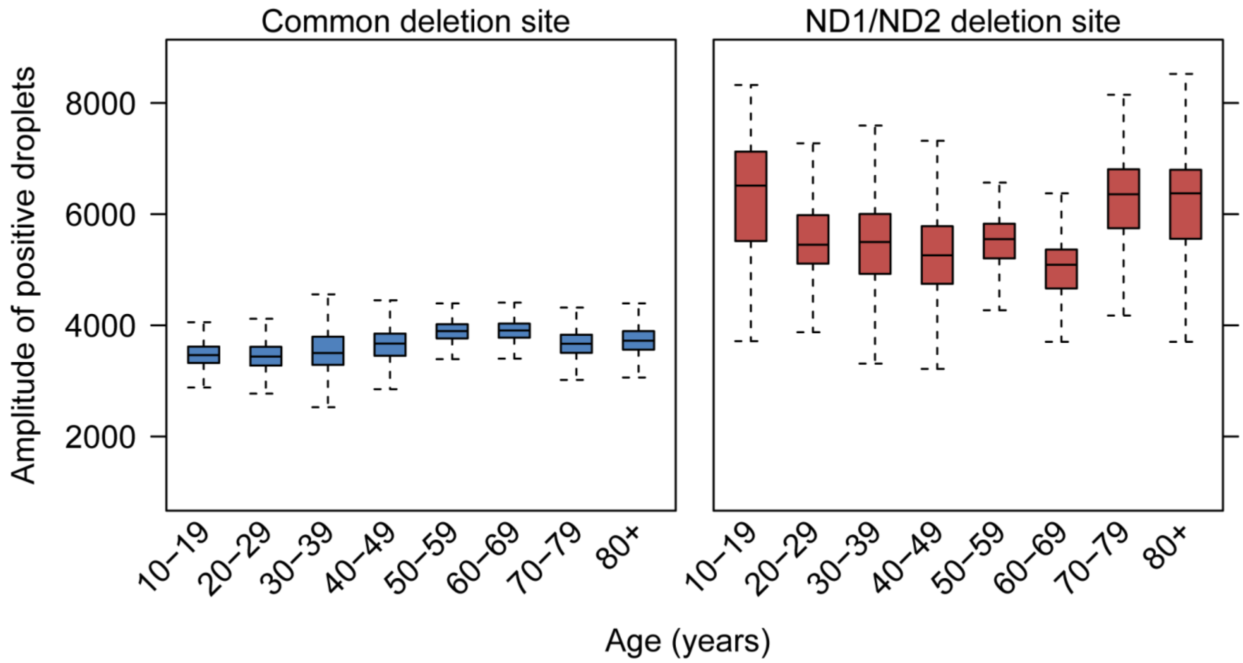

Figure S7. Distribution of positive droplets from ddPCR of human brain. Box and whisker plot showing the cumulative distribution of positive droplets across all patients, binned by decade. The relative distributions predict that the ND1/ND2 site accumulates a greater diversity of deletions than the common deletion site, but that the total diversity at both sites does not change appreciably with age.

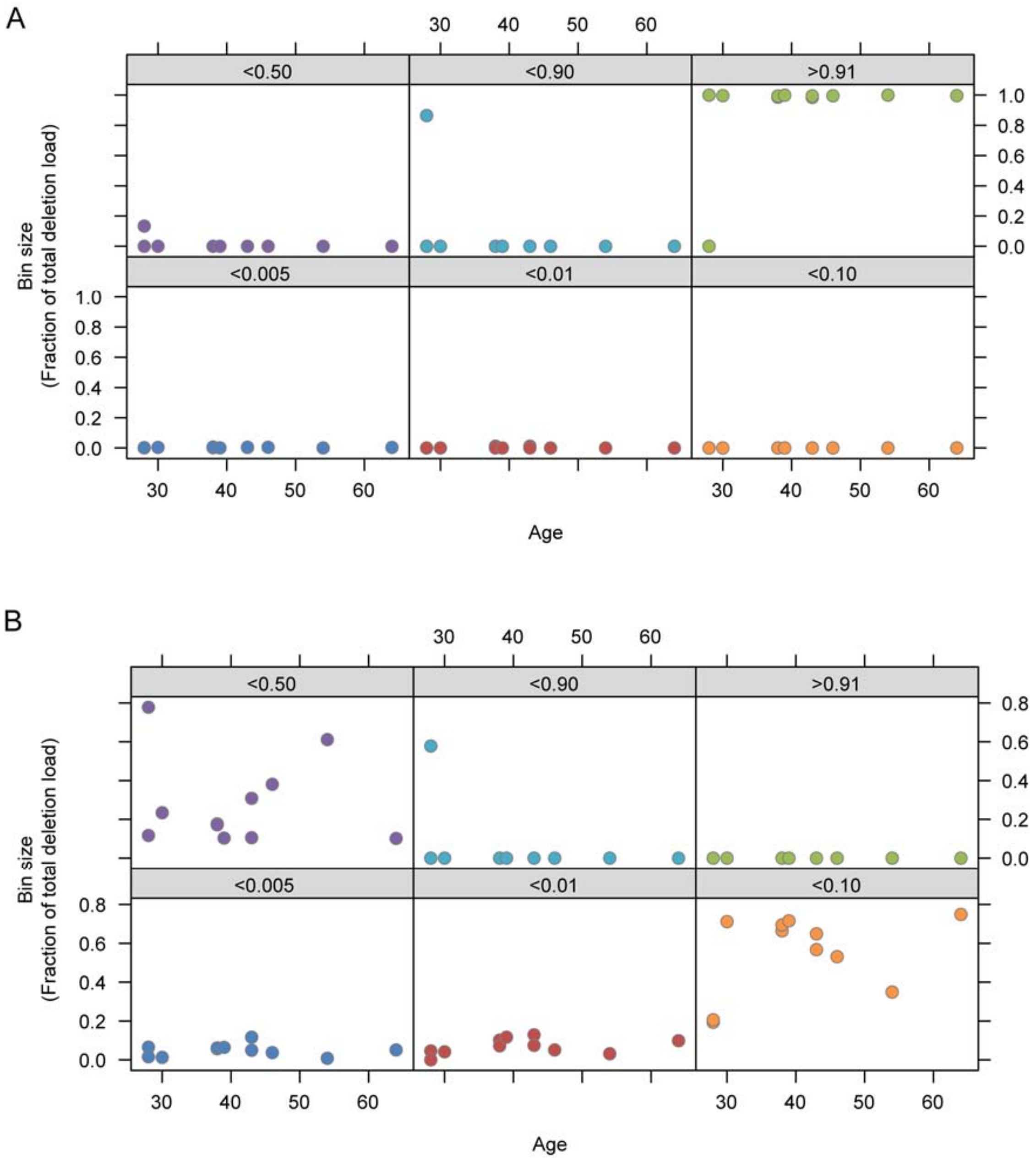

Figure S8. High resolution analysis of deletion dynamics. Unique deletions from each patient are binned into classes according to their relative frequency in the deletion pool. The cumulative fraction of each bin within the deletion pool is then plotted as a function of age for the **(A)** common and **(B)** ND1/ND2 site. At the common site, the deletion pool is dominated by a single species representing the canonical common deletion. At the ND1/ND2 site, deletions that individually contribute between 1 and 10% of the total number of deletions comprise the largest class within the deletion pool. Importantly, the relative size of the various deletion classes (or bins) do not shift with respect to age as might be expected with an age-associated accelerated rate of *de novo* deletion accumulation.

References

Edgar D, Shabalina I, Camara Y, Wredenberg A, Calvaruso MA, Nijtmans L, Nedergaard J, Cannon B, Larsson NG , Trifunovic A (2009). Random point mutations with major effects on protein-coding genes are the driving force behind premature aging in mtDNA mutator mice. *Cell Metab*. **10**, 131-138.

Krishnan KJ, Reeve AK, Samuels DC, Chinnery PF, Blackwood JK, Taylor RW, Wanrooij S, Spelbrink JN, Lightowlers RN , Turnbull DM (2008). What causes mitochondrial DNA deletions in human cells? *Nat Genet*. **40**, 275-279.

Kujoth GC, Hiona A, Pugh TD, Someya S, Panzer K, Wohlgemuth SE, Hofer T, Seo AY, Sullivan R, Jobling WA, Morrow JD, Van Remmen H, Sedivy JM, Yamasoba T, Tanokura M, Weindruch R, Leeuwenburgh C , Prolla TA (2005). Mitochondrial DNA mutations, oxidative stress, and apoptosis in mammalian aging. *Science*. **309**, 481-484.

Pinheiro LB, Coleman VA, Hindson CM, Herrmann J, Hindson BJ, Bhat S , Emslie KR (2012). Evaluation of a droplet digital polymerase chain reaction format for DNA copy number quantification. *Anal Chem*. **84**, 1003-1011.

Vermulst M, Bielas JH, Kujoth GC, Ladiges WC, Rabinovitch PS, Prolla TA , Loeb LA (2007). Mitochondrial point mutations do not limit the natural lifespan of mice. *Nat Genet*. **39**, 540-543.

Vermulst M, Wanagat J, Kujoth GC, Bielas JH, Rabinovitch PS, Prolla TA , Loeb LA (2008). DNA deletions and clonal mutations drive premature aging in mitochondrial mutator mice. *Nat Genet*. **40**, 392-394.
