# Supplementary material for: Targeted enrichment and high-resolution digital profiling of mitochondrial DNA deletions in human brain
Source: Aging Cell. 2013 Sep 11;13(1):29–38. doi: 10.1111/acel.12146 (PMC4068027; doi:10.1111/acel.12146)
Supplement: Supplementary file 2 — Data S1. Supplementary notes, methods and figures. Data S1A. Optimization of amplification factors. Data S1B. Residual amplification bias. Data S1C. Biases inherent in downstream applications. Data S1D. Validation in biological samples. Data S1E. Validation of sampling and analysis. Data S1F. Disruption of droplet emulsions Data S2. Tables of all accepted deletions for each patient. Sequence data deposited in the NCBI Sequence Read Archive under project accession number SRP027401. [file acel0013-0029-sd2.pdf]

Common site

Accepted distinct deletions

PATIENT: P01
TOTAL NUMBER OF READS ACCEPTED: 132698
UNIQUE DELETIONS ACCEPTED: 3

BP1	BP2	Length	Reads
8449	13414	4965	31
8482	13460	4978	132614
8474	13456	4982	53

PATIENT: P02
TOTAL NUMBER OF READS ACCEPTED: 134595
UNIQUE DELETIONS ACCEPTED: 5

BP1	BP2	Length	Reads
8449	13414	4965	202
8482	13460	4978	116346
8472	13459	4987	17973
8415	13422	5007	23
8437	13459	5022	51

PATIENT: P03
TOTAL NUMBER OF READS ACCEPTED: 480769
UNIQUE DELETIONS ACCEPTED: 8

BP1	BP2	Length	Reads
8449	13414	4965	1195
8455	13431	4976	64
8482	13460	4978	473294
8474	13456	4982	134
8436	13443	5007	243
8416	13426	5010	3217
8414	13436	5022	2570
8411	13466	5055	52

PATIENT: P04
TOTAL NUMBER OF READS ACCEPTED: 572381
UNIQUE DELETIONS ACCEPTED: 9

BP1	BP2	Length	Reads
8476	13432	4956	730
8476	13440	4964	15

8449	13414	4965	240
8455	13431	4976	17
8482	13460	4978	570146
8474	13456	4982	592
8415	13422	5007	547
8437	13459	5022	82
8427	13463	5036	12

PATIENT: P05
TOTAL NUMBER OF READS ACCEPTED: 546444
UNIQUE DELETIONS ACCEPTED: 6

BP1	BP2	Length	Reads
8449	13414	4965	30
8455	13431	4976	19
8482	13460	4978	538993
8436	13443	5007	935
8416	13426	5010	3288
8414	13436	5022	3179

PATIENT: P06
TOTAL NUMBER OF READS ACCEPTED: 664988
UNIQUE DELETIONS ACCEPTED: 6

BP1	BP2	Length	Reads
8487	13450	4963	267
8469	13438	4969	853
8455	13431	4976	45
8482	13460	4978	660825
8435	13415	4980	263
8436	13443	5007	2735

PATIENT: P07
TOTAL NUMBER OF READS ACCEPTED: 392895
UNIQUE DELETIONS ACCEPTED: 3

BP1	BP2	Length	Reads
8455	13431	4976	75
8482	13460	4978	392621
8474	13456	4982	199

PATIENT: P08
TOTAL NUMBER OF READS ACCEPTED: 490841

UNIQUE DELETIONS ACCEPTED: 4

BP1	BP2	Length	Reads
8455	13431	4976	42
8482	13460	4978	488252
8474	13456	4982	157
8436	13443	5007	2390

PATIENT: P09
TOTAL NUMBER OF READS ACCEPTED: 541796
UNIQUE DELETIONS ACCEPTED: 5

BP1	BP2	Length	Reads
8455	13431	4976	17
8482	13460	4978	539258
8474	13456	4982	163
8415	13422	5007	2336
8441	13449	5008	22

PATIENT: P10
TOTAL NUMBER OF READS ACCEPTED: 326130
UNIQUE DELETIONS ACCEPTED: 3

BP1	BP2	Length	Reads
8482	13460	4978	326024
8474	13456	4982	86
8436	13443	5007	20

PATIENT: P11
TOTAL NUMBER OF READS ACCEPTED: 328146
UNIQUE DELETIONS ACCEPTED: 6

BP1	BP2	Length	Reads
8453	13430	4977	87
8482	13460	4978	326937
8474	13456	4982	106
8416	13426	5010	889
8427	13462	5035	116
8411	13447	5036	11

ND1/ND2 site
Accepted distinct deletions

PATIENT: P01
TOTAL NUMBER OF READS ACCEPTED: 6854
UNIQUE DELETIONS ACCEPTED: 41

BP1	BP2	Length	Reads
3895	4352	457	23
3900	4462	562	37
3874	4438	564	27
3807	4391	584	23
3837	4485	648	25
3774	4425	651	72
3851	4539	688	10
3666	4360	694	56
3642	4355	713	12
3693	4428	735	57
3747	4485	738	83
3776	4516	740	13
3758	4501	743	69
3717	4486	769	71
3588	4358	770	14
3627	4398	771	35
3771	4551	780	3963
3575	4357	782	149
3638	4433	795	23
3708	4504	796	39
3558	4376	818	13
3659	4482	823	74
3613	4438	825	33
3552	4396	844	17
3614	4460	846	45
3650	4502	852	33
3582	4439	857	801
3608	4465	857	20
3610	4486	876	19
3576	4459	883	111
3728	4617	889	15
3533	4427	894	22
3587	4491	904	69
3547	4456	909	48
3570	4486	916	549
3679	4610	931	26
3549	4510	961	10
3548	4535	987	22

3545	4545	1000	79
3582	4618	1036	21
3547	4594	1047	26

PATIENT: P02
TOTAL NUMBER OF READS ACCEPTED: 20120
UNIQUE DELETIONS ACCEPTED: 19

BP1	BP2	Length	Reads
3895	4352	457	1196
3748	4403	655	1458
3719	4418	699	16
3568	4352	784	69
3704	4523	819	57
3705	4547	842	11
3675	4528	853	20
3630	4485	855	41
3582	4439	857	3313
3613	4479	866	4142
3718	4585	867	689
3580	4468	888	18
3546	4438	892	11
3570	4480	910	17
3610	4567	957	803
3640	4598	958	18
3549	4510	961	27
3660	4641	981	14
3551	4543	992	8200

PATIENT: P03
TOTAL NUMBER OF READS ACCEPTED: 28116
UNIQUE DELETIONS ACCEPTED: 88

BP1	BP2	Length	Reads
3895	4352	457	28
3855	4389	534	78
3835	4373	538	56
3896	4440	544	170
3783	4356	573	163
3822	4395	573	123
3807	4391	584	154
3874	4458	584	219
3853	4438	585	30
3799	4393	594	118
3836	4438	602	58

3852	4458	606	33
3783	4395	612	89
3824	4436	612	40
3853	4476	623	152
3852	4485	633	105
3822	4456	634	730
3783	4422	639	135
3799	4438	639	110
3824	4477	653	139
3783	4438	655	100
3683	4368	685	128
3662	4356	694	231
3784	4481	697	198
3835	4539	704	16
3878	4586	708	23
3747	4459	712	138
3901	4622	721	46
3710	4438	728	45
3833	4565	732	137
3691	4439	748	1551
3853	4606	753	21
3631	4385	754	30
3588	4358	770	156
3710	4484	774	1660
3612	4388	776	92
3642	4422	780	198
3575	4357	782	1067
3683	4468	785	77
3705	4497	792	459
3630	4430	800	213
3825	4632	807	45
3546	4356	810	191
3671	4484	813	548
3642	4459	817	368
3613	4438	825	541
3707	4533	826	2371
3530	4357	827	83
3630	4458	828	116
3783	4618	835	108
3705	4547	842	111
3669	4513	844	291
3681	4525	844	244
3763	4607	844	96
3613	4458	845	145
3691	4537	846	48
3696	4552	856	367
3582	4439	857	2965

3671	4532	861	481
3552	4415	863	331
3530	4396	866	11
3571	4438	867	1810
3614	4481	867	568
3587	4459	872	212
3721	4608	887	61
3580	4468	888	17
3599	4490	891	187
3670	4561	891	16
3543	4437	894	187
3576	4480	904	44
3587	4491	904	1131
3659	4565	906	314
3530	4438	908	126
3592	4517	925	15
3529	4459	930	155
3690	4628	938	18
3546	4485	939	231
3586	4525	939	1107
3534	4484	950	219
3545	4501	956	484
3611	4568	957	122
3666	4631	965	11
3552	4534	982	1510
3637	4624	987	74
3599	4592	993	49
3567	4563	996	555
3529	4534	1005	83
3613	4618	1005	33

PATIENT: P04
TOTAL NUMBER OF READS ACCEPTED: 5598
UNIQUE DELETIONS ACCEPTED: 32

BP1	BP2	Length	Reads
3895	4352	457	94
3901	4386	485	69
3871	4434	563	167
3836	4432	596	36
3811	4428	617	69
3804	4427	623	31
3742	4438	696	30
3706	4423	717	405
3743	4460	717	67
3710	4438	728	11

3644	4378	734	64
3771	4551	780	514
3568	4352	784	88
3702	4515	813	128
3569	4394	825	57
3685	4510	825	285
3701	4534	833	355
3531	4377	846	50
3614	4460	846	97
3705	4554	849	366
3643	4499	856	201
3582	4439	857	1311
3575	4440	865	89
3580	4468	888	24
3598	4490	892	46
3641	4539	898	126
3587	4491	904	386
3570	4480	910	18
3567	4515	948	39
3549	4510	961	18
3555	4579	1024	250
3575	4619	1044	107

PATIENT: P05
TOTAL NUMBER OF READS ACCEPTED: 13028
UNIQUE DELETIONS ACCEPTED: 64

BP1	BP2	Length	Reads
3895	4352	457	24
3885	4356	471	39
3896	4384	488	63
3897	4422	525	72
3898	4436	538	122
3783	4355	572	16
3885	4458	573	461
3796	4370	574	24
3807	4391	584	89
3879	4483	604	132
3872	4484	612	203
3837	4461	624	89
3852	4485	633	242
3871	4507	636	40
3836	4484	648	24
3705	4359	654	135
3801	4461	660	121
3783	4457	674	240

3835	4514	679	158
3677	4369	692	112
3693	4399	706	110
3771	4478	707	131
3637	4354	717	90
3707	4428	721	312
3895	4619	724	42
3882	4616	734	13
3613	4356	743	202
3758	4501	743	277
3691	4438	747	308
3631	4385	754	30
3771	4539	768	372
3582	4356	774	208
3727	4510	783	65
3568	4352	784	36
3566	4360	794	706
3581	4376	795	19
3637	4438	801	154
3712	4516	804	330
3689	4494	805	12
3579	4386	807	143
3666	4476	810	186
3569	4394	825	366
3613	4438	825	226
3530	4356	826	30
3710	4539	829	114
3658	4510	852	239
3630	4485	855	173
3582	4439	857	2303
3535	4401	866	22
3613	4479	866	106
3610	4486	876	86
3697	4579	882	38
3580	4468	888	618
3610	4499	889	382
3598	4489	891	271
3666	4565	899	117
3576	4480	904	48
3587	4491	904	1250
3612	4539	927	97
3691	4618	927	64
3683	4612	929	13
3575	4530	955	227
3644	4616	972	47
3573	4621	1048	39

PATIENT: P06
TOTAL NUMBER OF READS ACCEPTED: 19282
UNIQUE DELETIONS ACCEPTED: 58

BP1	BP2	Length	Reads
3874	4356	482	53
3896	4395	499	291
3836	4354	518	226
3896	4440	544	183
3896	4464	568	220
3872	4457	585	248
3885	4479	594	279
3872	4484	612	197
3852	4485	633	104
3871	4506	635	71
3799	4438	639	299
3782	4423	641	381
3901	4542	641	40
3836	4484	648	356
3825	4479	654	29
3820	4485	665	83
3839	4509	670	100
3800	4481	681	313
3899	4580	681	436
3754	4438	684	156
3771	4478	707	498
3899	4613	714	102
3822	4566	744	165
3856	4603	747	59
3591	4359	768	59
3692	4460	768	949
3568	4352	784	18
3784	4568	784	1315
3697	4484	787	115
3746	4539	793	257
3637	4438	801	63
3671	4484	813	64
3702	4515	813	799
3569	4394	825	339
3712	4538	826	396
3697	4525	828	562
3675	4512	837	42
3582	4439	857	3313
3648	4515	867	317
3580	4468	888	281
3598	4489	891	95

3546	4438	892	707
3631	4525	894	130
3576	4480	904	67
3587	4491	904	1520
3605	4510	905	167
3568	4486	918	898
3710	4634	924	57
3691	4618	927	85
3640	4570	930	693
3618	4551	933	167
3666	4602	936	80
3541	4484	943	342
3534	4479	945	56
3547	4508	961	280
3586	4596	1010	71
3545	4600	1055	67
3529	4619	1090	22

PATIENT: P07
TOTAL NUMBER OF READS ACCEPTED: 39039
UNIQUE DELETIONS ACCEPTED: 84

BP1	BP2	Length	Reads
3895	4352	457	586
3889	4356	467	20
3837	4353	516	265
3896	4423	527	131
3825	4360	535	76
3878	4427	549	740
3889	4438	549	189
3824	4386	562	203
3783	4355	572	257
3898	4476	578	143
3807	4391	584	11
3895	4491	596	523
3824	4422	598	526
3889	4490	601	530
3837	4456	619	734
3824	4456	632	282
3785	4428	643	244
3852	4500	648	323
3783	4439	656	695
3820	4485	665	207
3814	4483	669	231
3785	4461	676	722
3799	4478	679	27

3711	4391	680	260
3841	4526	685	154
3795	4485	690	200
3784	4476	692	11
3742	4438	696	578
3675	4376	701	132
3656	4358	702	193
3785	4493	708	412
3730	4440	710	257
3668	4379	711	545
3838	4554	716	477
3807	4530	723	15
3710	4437	727	1012
3747	4485	738	457
3758	4501	743	14
3774	4517	743	404
3666	4431	765	345
3693	4461	768	4042
3710	4484	774	1808
3771	4551	780	243
3800	4580	780	143
3575	4357	782	1174
3699	4484	785	474
3666	4456	790	825
3571	4375	804	364
3712	4516	804	2251
3630	4438	808	96
3543	4354	811	539
3802	4614	812	128
3671	4484	813	96
3700	4514	814	773
3578	4397	819	14
3569	4394	825	495
3613	4438	825	326
3630	4458	828	32
3666	4499	833	757
3705	4547	842	17
3760	4612	852	58
3675	4528	853	22
3712	4565	853	249
3582	4439	857	2674
3613	4479	866	15
3705	4579	874	114
3666	4542	876	611
3580	4468	888	830
3598	4490	892	303
3595	4495	900	129

3587	4491	904	969
3530	4439	909	777
3576	4485	909	3338
3700	4615	915	172
3543	4476	933	123
3534	4484	950	29
3611	4568	957	15
3549	4510	961	696
3641	4602	961	19
3659	4632	973	11
3650	4625	975	22
3587	4621	1034	15
3576	4625	1049	11
3547	4607	1060	109

PATIENT: P08
TOTAL NUMBER OF READS ACCEPTED: 73573
UNIQUE DELETIONS ACCEPTED: 57

BP1	BP2	Length	Reads
3898	4355	457	603
3896	4395	499	313
3897	4438	541	333
3835	4380	545	59
3783	4356	573	1233
3900	4509	609	1250
3888	4506	618	54
3837	4476	639	1307
3799	4439	640	157
3846	4486	640	10
3884	4539	655	173
3799	4462	663	5743
3871	4539	668	397
3890	4561	671	354
3799	4479	680	1188
3895	4579	684	150
3666	4354	688	8021
3677	4369	692	11
3822	4515	693	7042
3655	4381	726	267
3710	4438	728	1656
3747	4485	738	14675
3666	4406	740	125
3853	4606	753	687
3718	4479	761	488
3691	4458	767	281

3712	4483	771	2261
3847	4620	773	41
3784	4565	781	982
3568	4352	784	65
3840	4642	802	20
3712	4516	804	2219
3632	4457	825	15
3643	4477	834	105
3642	4485	843	1691
3613	4458	845	865
3675	4528	853	12
3660	4515	855	2189
3582	4439	857	2955
3613	4479	866	169
3575	4459	884	1427
3693	4581	888	3148
3598	4489	891	506
3561	4456	895	2634
3587	4491	904	413
3574	4486	912	670
3539	4457	918	358
3588	4506	918	349
3710	4634	924	956
3529	4459	930	646
3543	4476	933	669
3529	4480	951	405
3611	4568	957	11
3549	4510	961	16
3529	4516	987	187
3545	4545	1000	999
3531	4533	1002	13

PATIENT: P09
TOTAL NUMBER OF READS ACCEPTED: 35833
UNIQUE DELETIONS ACCEPTED: 49

BP1	BP2	Length	Reads
3895	4352	457	150
3872	4354	482	60
3836	4356	520	387
3899	4425	526	122
3825	4360	535	376
3896	4440	544	369
3890	4456	566	250
3836	4458	622	51
3797	4423	626	101

3895	4541	646	10
3837	4485	648	197
3771	4446	675	2374
3690	4366	676	685
3799	4479	680	777
3742	4438	696	261
3771	4475	704	366
3835	4539	704	9637
3710	4423	713	312
3807	4530	723	215
3706	4431	725	50
3853	4616	763	171
3710	4484	774	689
3680	4460	780	669
3708	4497	789	842
3561	4354	793	241
3840	4642	802	14
3690	4506	816	626
3613	4438	825	661
3710	4539	829	92
3639	4473	834	51
3666	4508	842	148
3642	4485	843	622
3780	4628	848	12
3703	4556	853	20
3582	4439	857	2612
3671	4539	868	869
3580	4468	888	4004
3598	4490	892	43
3534	4438	904	2259
3582	4491	909	1714
3709	4622	913	122
3551	4485	934	496
3690	4628	938	14
3672	4629	957	10
3535	4523	988	44
3596	4601	1005	13
3613	4618	1005	31
3535	4558	1023	1637
3576	4618	1042	357

PATIENT: P10
TOTAL NUMBER OF READS ACCEPTED: 56656
UNIQUE DELETIONS ACCEPTED: 24

BP1	BP2	Length	Reads

3895	4352	457	46
3854	4354	500	30
3899	4427	528	509
3885	4438	553	2038
3697	4386	689	75
3631	4385	754	1487
3710	4484	774	285
3784	4558	774	2496
3568	4352	784	18
3706	4500	794	622
3642	4438	796	320
3840	4642	802	27
3671	4484	813	4600
3613	4438	825	16
3582	4439	857	10436
3610	4486	876	20
3598	4489	891	2221
3587	4491	904	2347
3569	4478	909	3968
3723	4632	909	353
3573	4500	927	24201
3530	4480	950	206
3534	4532	998	320
3545	4545	1000	15

PATIENT: P11
TOTAL NUMBER OF READS ACCEPTED: 38175
UNIQUE DELETIONS ACCEPTED: 67

BP1	BP2	Length	Reads
3895	4352	457	102
3882	4407	525	303
3898	4430	532	180
3798	4357	559	649
3874	4437	563	111
3783	4352	569	325
3799	4393	594	313
3853	4456	603	22
3852	4465	613	423
3820	4439	619	166
3852	4485	633	310
3900	4536	636	2237
3784	4436	652	155
3799	4456	657	11
3837	4503	666	399
3783	4458	675	301

3884	4576	692	685
3742	4438	696	530
3780	4476	696	499
3835	4539	704	544
3743	4460	717	276
3764	4481	717	136
3807	4530	723	730
3742	4479	737	263
3613	4356	743	172
3710	4458	748	77
3824	4579	755	76
3696	4457	761	736
3691	4458	767	1803
3710	4485	775	489
3643	4422	779	229
3771	4551	780	609
3572	4356	784	228
3699	4485	786	1503
3691	4486	795	737
3637	4438	801	337
3712	4516	804	1274
3666	4482	816	3880
3530	4355	825	901
3613	4438	825	1215
3701	4532	831	1807
3659	4499	840	171
3705	4547	842	572
3613	4458	845	106
3666	4512	846	574
3705	4559	854	440
3582	4439	857	1815
3594	4458	864	23
3571	4438	867	643
3613	4485	872	58
3672	4544	872	657
3533	4411	878	65
3605	4496	891	271
3598	4490	892	45
3665	4565	900	415
3685	4592	907	205
3530	4439	909	394
3572	4481	909	206
3582	4491	909	2603
3706	4617	911	200
3659	4591	932	104
3549	4510	961	27
3585	4554	969	1852

3545	4545	1000	839
3573	4621	1048	120
3553	4636	1083	17
3530	4618	1088	10
