# Supplementary figures and images for: Targeted enrichment and high-resolution digital profiling of mitochondrial DNA deletions in human brain
Source: Aging Cell. 2013 Sep 11;13(1):29–38. doi: 10.1111/acel.12146 (PMC4068027; doi:10.1111/acel.12146)

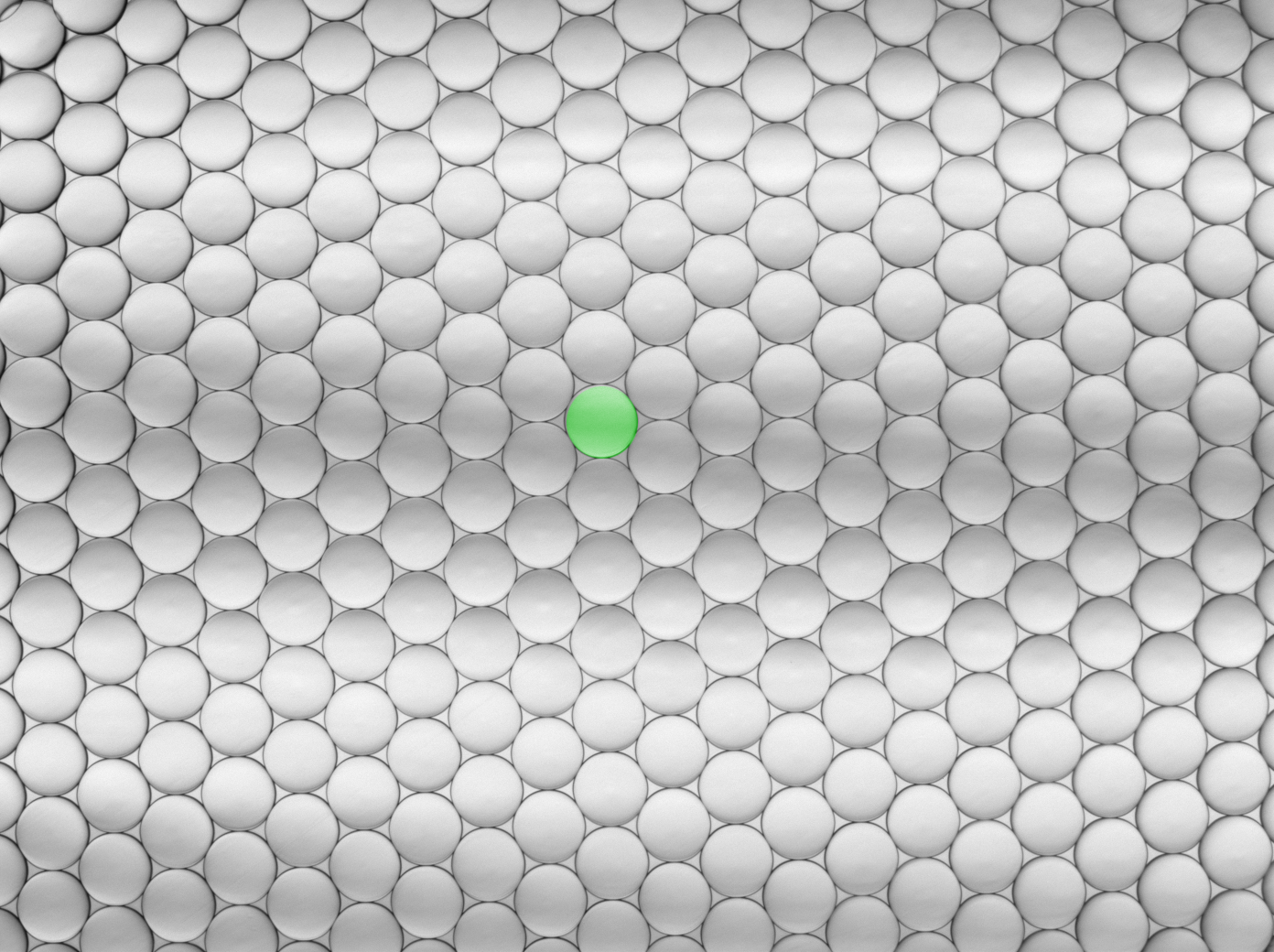

Supplement: Supplementary file 3 [file acel0013-0029-sd3.tif]
